# Intermittent hypoxia in a mouse model of apnea of prematurity leads to a retardation of cerebellar development and long-term functional deficits

**DOI:** 10.1186/s13578-022-00869-5

**Published:** 2022-09-06

**Authors:** S. Leroux, A. Rodriguez-Duboc, A. Arabo, M. Basille-Dugay, D. Vaudry, D. Burel

**Affiliations:** 1 Laboratory of Neuronal and Neuroendocrine Communication and Differentiation, Institute for Research and Innovation in Biomedicine (IRIB), Normandie Univ, UNIROUEN, INSERM U1239, 76000 Rouen, France; 2 Cancer and brain genomics, Institute for Research and Innovation in Biomedicine (IRIB), Normandie Univ, UNIROUEN, INSERM U1245, 76000 Rouen, France; 3 Faculty of Sciences, Experimental Investigation - Service Ressources Biologiques (SRB), Institute for Research and Innovation in Biomedicine (IRIB), Normandie University, UNIROUEN, 76000 Rouen, France; 4 Regional Platform for Cell Imaging of Normandy (PRIMACEN), Normandie University, UNIROUEN, 76000 Rouen, France

**Keywords:** Apnea of prematurity, Intermittent hypoxia, Cerebellum, Purkinje cells

## Abstract

**Background:**

Apnea of prematurity (AOP) is caused by respiratory control immaturity and affects nearly 50% of premature newborns. This pathology induces perinatal intermittent hypoxia (IH), which leads to neurodevelopmental disorders. The impact on the brain has been well investigated. However, despite its functional importance and immaturity at birth, the involvement of the cerebellum remains poorly understood. Therefore, this study aims to identify the effects of IH on cerebellar development using a mouse model of AOP consisting of repeated 2-min cycles of hypoxia and reoxygenation over 6 h and for 10 days starting on postnatal day 2 (P2).

**Results:**

At P12, IH-mice cerebella present higher oxidative stress associated with delayed maturation of the cerebellar cortex and decreased dendritic arborization of Purkinje cells. Moreover, mice present with growth retardation and motor disorders. In response to hypoxia, the developing cerebellum triggers compensatory mechanisms resulting in the unaltered organization of the cortical layers from P21 onwards. Nevertheless, some abnormalities remain in adult Purkinje cells, such as the dendritic densification, the increase in afferent innervation, and axon hypomyelination. Moreover, this compensation seems insufficient to allow locomotor recovery because adult mice still show motor impairment and significant disorders in spatial learning.

**Conclusions:**

All these findings indicate that the cerebellum is a target of intermittent hypoxia through alterations of developmental mechanisms leading to long-term functional deficits. Thus, the cerebellum could contribute, like others brain structures, to explaining the pathophysiology of AOP.

**Supplementary Information:**

The online version contains supplementary material available at 10.1186/s13578-022-00869-5.

## Background

Apnea of prematurity (AOP) is a developmental disorder characterized by a sudden cessation of breathing, lasting at least 20 s and/or associated with bradycardia or oxygen desaturation [1]. This condition affects at least 50% of premature infants and nearly 100% of extremely preterm neonates, born before 29 weeks of gestation [2]. Although it has been shown that apneas are mostly due to a physiological immaturity of respiratory control, the pathogenesis and neurological consequences of this disease remain unclear [3].

Nonetheless, as improvements in healthcare have helped reduce mortality in recent years, long-term deficits following AOP are now noticeable. Indeed, it has been shown that AOP was associated with an increased risk of periventricular leukomalacia [4, 5]. Moreover, a correlation has been established between apnea duration and neurodevelopmental disorders, including language, cognitive, and motor impairments [6]. These deficits appear to reflect the severity of the apnea, with short respiratory arrests being inconsequential and longer ones deleterious [7].

Several animal models of intermittent hypoxia (IH) have been developed to better understand the pathogenesis and outcomes of AOP in humans [8–10]. The repeated cycles of hypoxia and reoxygenation that characterize this pathology have been shown to induce cellular damage and oxidative stress, leading to central nervous system injuries. Indeed, Cai et al. [11] demonstrated oligodendrocyte and synaptic alterations in the striatum and corpus callosum following perinatal IH. Moreover, decreased long-term potentiation and memory disorders have also been observed [9, 10].

However, despite the increasing number of studies trying to assess the effects of AOP on the brain, very few investigate its impact on the rest of the nervous system, notably the cerebellum which is particularly vulnerable. Indeed, the cerebellar cortex is immature at birth [12] and consists of 4 layers that undergo an important maturation process from the last trimester of pregnancy and well into the postnatal period. In humans, the granular cell precursors (GCP) first proliferate in the external granular layer (EGL), which reaches its peak thickness between the 20th and 30th weeks of gestation. These neuron precursors then migrate through the molecular layer (ML) thanks to the Bergmann glia, cross the Purkinje cell layer (PL), and differentiate to form the internal granular layer (IGL) [13]. In the meantime, Purkinje cells develop their dendritic arborization. This allows for the establishment of synaptic contacts with ascending granule cell axons, called parallel fibers, and for the enlargement of the ML. As a result, these neurons receive indirect information from mossy fibers and integrate it along with data directly supplied by the climbing fibers from the olivary nucleus.

Thus, the cerebellum controls various motor behaviors such as equilibrium or motor coordination, but also higher functions such as language, learning, or spatial orientation [14–16]. It appears that most of these processes are altered in children affected by AOP [6, 7]. In fact, Sathyanesan et al. [17] demonstrated that chronic perinatal hypoxia induces persistent motor coordination disorders and learning deficits. These data suggest that perinatal incidents such as apnea could affect cerebellar development and lead to behavioral impairments. Based on this hypothesis, it has been shown that IH in rodents causes Purkinje cell alterations, hypomyelination, and oxidative stress [8, 11, 18, 19].

In this work, we decided to analyze cerebellar histology during development, as well as the behavior of young and adult mice in a model of AOP. We first determined the effect of intermittent hypoxia on oxidative stress, proliferation, apoptosis, and cortical layer organization. On a functional level, we investigated short and long-term motor and learning aptitudes to determine whether cellular defects were associated with behavioral disorders. Altogether, our results aim to correlate cerebellar alterations with the long-term deleterious consequences observed in preterm children having experienced AOP.

## Results

### Effects of perinatal intermittent hypoxia on cellular mechanisms of development

**Fig. 1:**
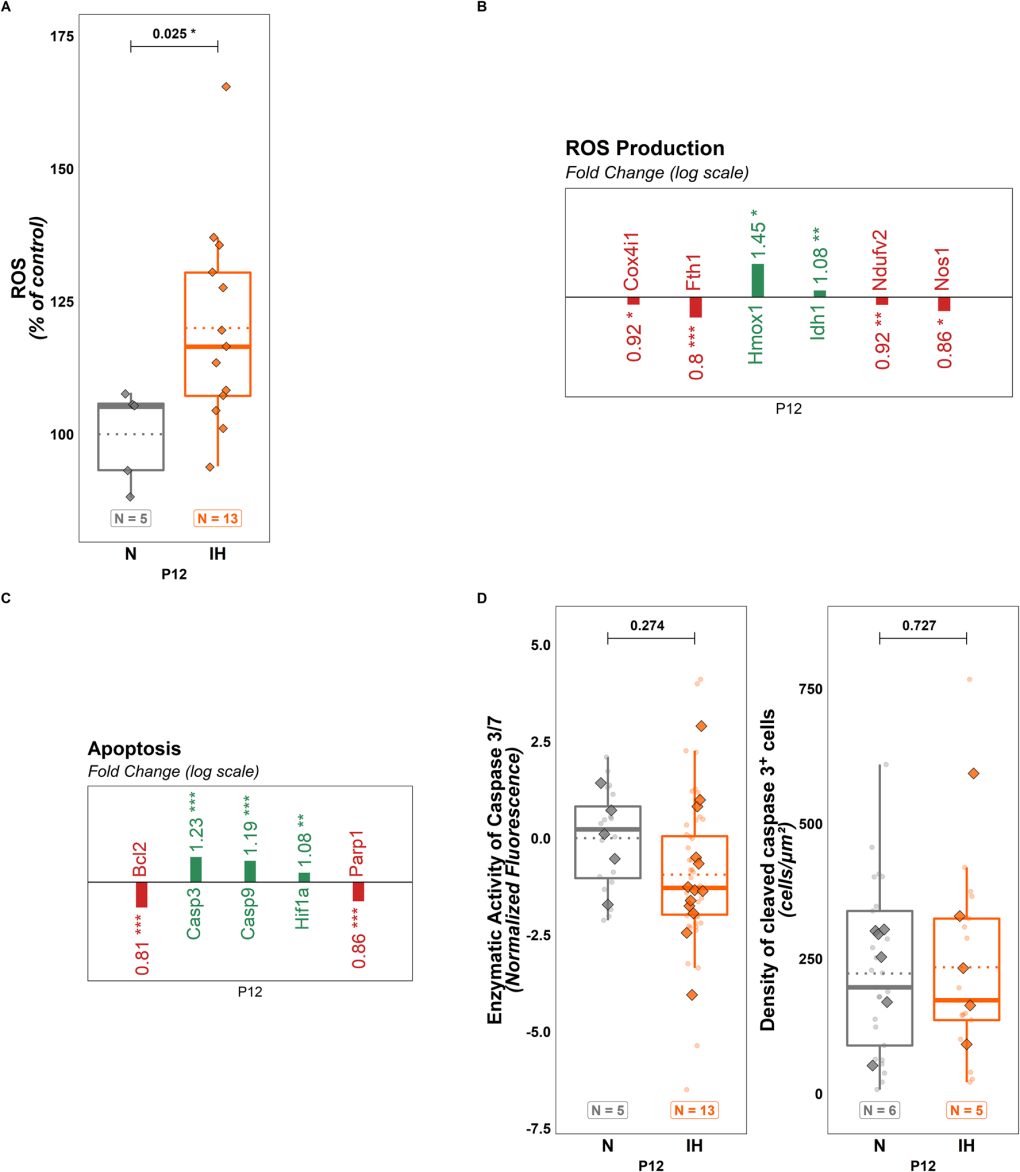
Effects of perinatal intermittent hypoxia on cellular mechanisms in P12 mice. **A** Quantification of ROS production in the whole cerebellum of control (N) or hypoxic (IH) P12 mice. **B** Significant RT-qPCR results reflecting the regulation of the expression of genes involved in the production of ROS in the whole cerebellum of control (N) or hypoxic (IH) P12 mice. **C** Significant RT-qPCR results reflecting the regulation of the expression of genes associated to apoptosis in the whole cerebellum of control (N) or hypoxic (IH) P12 mice. **D** Enzymatic activity of caspase-3/7 (left) and density of cleaved caspase 3 positive cells (right) in the whole cerebellum of control (N) or hypoxic (IH) P12 mice. For **B** and **C**, the number of animals was 15 for the N group and 10 for the IH group. For **A** and **D**, the total number of animals in each experimental group is indicated under the boxplots and represented by diamond shapes, while the transparent dots indicate individual data points. Exact p-values are indicated above the plot. IH: intermittent hypoxia condition; N: normoxia condition; P12: postnatal day 12; ROS: reactive oxygen species

In order to mimic apnea of prematurity, neonatal mice were exposed to cycles of hypoxia and reoxygenation (2 min/cycle, 6 h/day for 10 days, starting at the postnatal day 2 (P2)). To evaluate the oxidative stress induced by hypoxia in the cerebellum, the concentration of reactive oxygen species (ROS) produced in mice cerebella at the end of the apnea protocol was measured. We observed that IH induces an increase in ROS production in the whole cerebellum ($t\left(17\right)=-2.454;p=0.025;Ratio=0.833;C{I}_{95}=\left[\mathrm{0.713,0.975}\right]$; Fig. 1A). Moreover, our qPCR analysis shows that, of the 9 genes tested from the "ROS production" panel, 6 are differentially expressed at P12 after IH. More precisely, Cox4i1 and Ndufv2 are slightly under-expressed, Nos1 and Fth1 are more markedly under-expressed, and Hmox1 and Idh1 are over-expressed (Fig. 1B; see statistics in Additional file 1: Table S1).

**Fig. 2:**
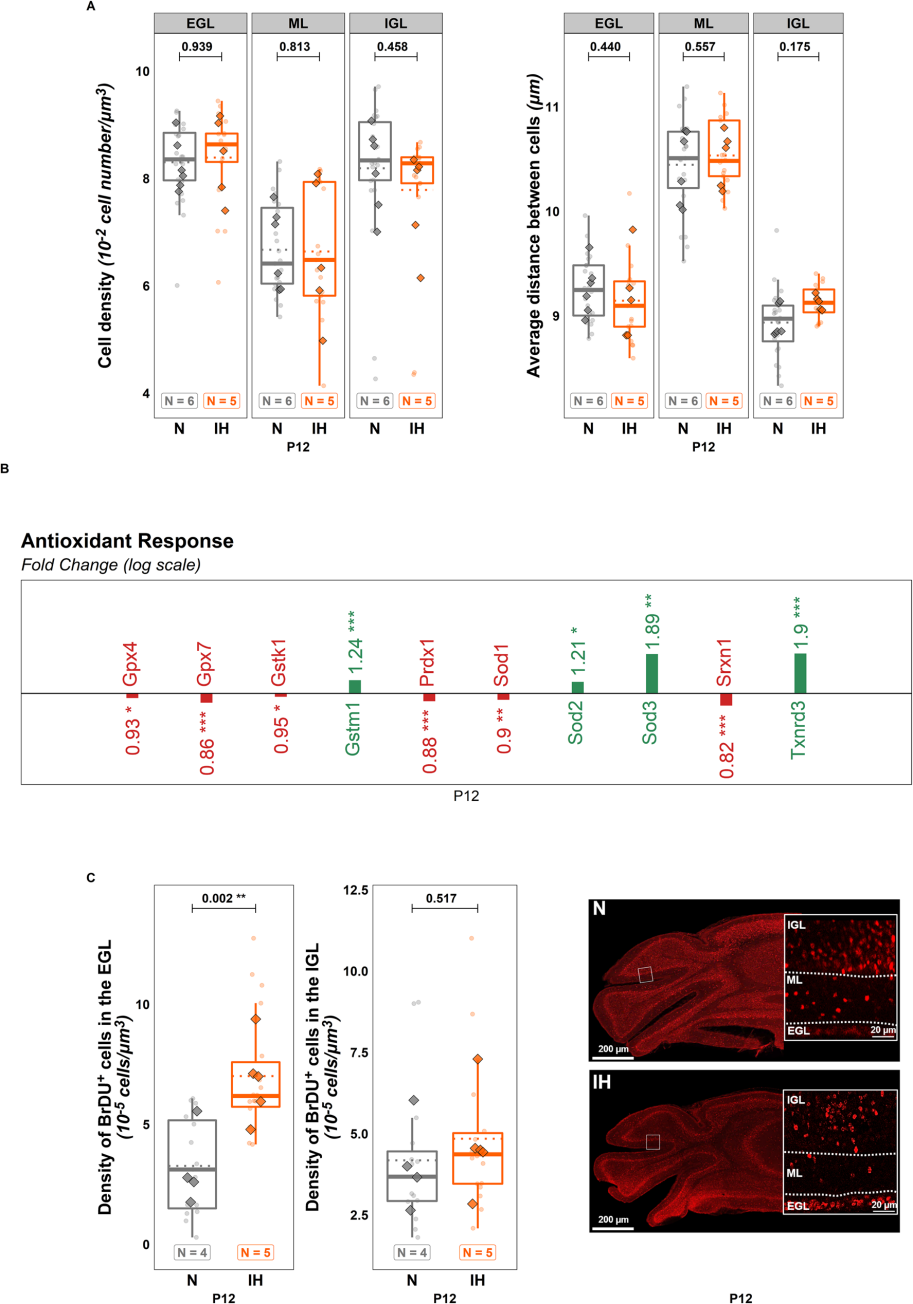
Cellular defense against oxidative stress during perinatal intermittent hypoxia in P12 mice. **A** Cell density (left) and average distance between cells (right) measured on DAPI-stained cerebellar slices of control (N) or hypoxic (IH) P12 mice. **B** Significant RT-qPCR results reflecting the regulation of the expression of genes involved in the antioxidant response to oxidative stress in the whole cerebellum of control (N) or hypoxic (IH) P12 mice. **C** Density of BrdU-immunoreactive cells in the EGL (left) and IGL (middle) and confocal images (right) illustrating the distribution of BrDU-positive cells in the cerebellar cortex in control (N) or hypoxic (IH) mice. For **B**, the number of animals was 15 for the N group and 10 for the IH group. For **A** and **C**, the total number of animals in each experimental group is indicated under the boxplots and represented by diamond shapes, while the transparent dots indicate individual data points. Exact p-values are indicated above the plot. BrdU: bromodeoxyuridine; EGL: external granular layer; IGL: internal granular layer; IH: intermittent hypoxia condition; N: normoxia condition; P12: postnatal day 12; ROS: reactive oxygen species

Concerning the "Apoptosis" panel, the expression of Casp3, Casp9, Hif1α was up-regulated while Bcl2 and Parp1 are under-expressed after IH at P12 (Fig. 1C, see statistics in Additional file 1: Table S1). However, this result is not reflected in terms of the enzymatic activity of caspases-3/7 or the density of cleaved caspase-3 positive cells (Fig. 1D). Similarly, DAPI labeling in the different cortical layers at P12 revealed no difference concerning the density of cells (spots per volume) (EGL: $t\left(120\right)= -0.076; p = 0.939; Ratio = 0.995; C{I}_{95}= \left[0.868, 1.14\right];$ ML: $t(120) = 0.237; p = 0.813; Ratio = 1.017; C{I}_{95} = [0.887, 1.166];$ IGL: $t(120) = 0.745; p = 0.458; Ratio = 1.053; C{I}_{95} = [0.918, 1.207];$ Fig. 2A) or in the average distance between cells (EGL: $t(120) = 0.775; p = 0.440; Ratio = 1.012; C{I}_{95} = [0.981, 1.044];$ ML: $t(120) = -0.589; p = 0.557; Ratio = 0.991; C{I}_{95} = [0.961, 1.022];$ IGL: $t(120) = -1.364; p = 0.175; Ratio = 0.979; C{I}_{95} = [0.949, 1.01];$ Fig. 2A).

Additionally, the effect of IH on the expression of genes implicated in the antioxidant response was studied. While some genes involved in redox homeostasis, such as Gpx4, Gsk1, Prdx1, Sod1 and Srxn1, are under-expressed after IH, thus reflecting cellular distress, other antioxidant genes like Sod 2, Sod 3, Txnrd3 and Gstm1 are over-expressed indicating the establishment of a defense system (Fig. 2B; see statistics in Additional file 1: Table S1). The impact of IH on GCP proliferation and migration was also analyzed by counting bromodeoxyuridine (BrDU)-positive cells (N group = 2 males and 2 females; IH group = 3 males and 2 females). P6 injection of BrDU helped us estimate the capacity of GCP to divide and then migrate to the IGL until P12. P12 administration revealed the immediate proliferation at the end of the IH protocol. Our results indicate that hypoxia did not change the number of BrDU-positive cells in the IGL, indicating no deficit of neuronal migration (Fig. 2C), but did increase BRDU-labeled precursors in the EGL ($t\left(31\right)=-3.295;p=0.002;Ratio=0.443;C{I}_{95}=\left[\mathrm{0.268,0.734}\right];$ Fig. 2C), demonstrating a higher proliferative activity.

### Effects of perinatal intermittent hypoxia on the structural organization of the developing cerebellum

Immunohistochemical studies were performed using DAPI and calbindin labeling at the end of the protocol (P12; N group = 3 males; IH group = 3 males and 1 female) or 9 days later (P21; N group = 1 male and 2 females; IH group = 2 males-1 female) to analyze the short and long-term effects of IH on cerebellar histology.

**Fig. 3:**
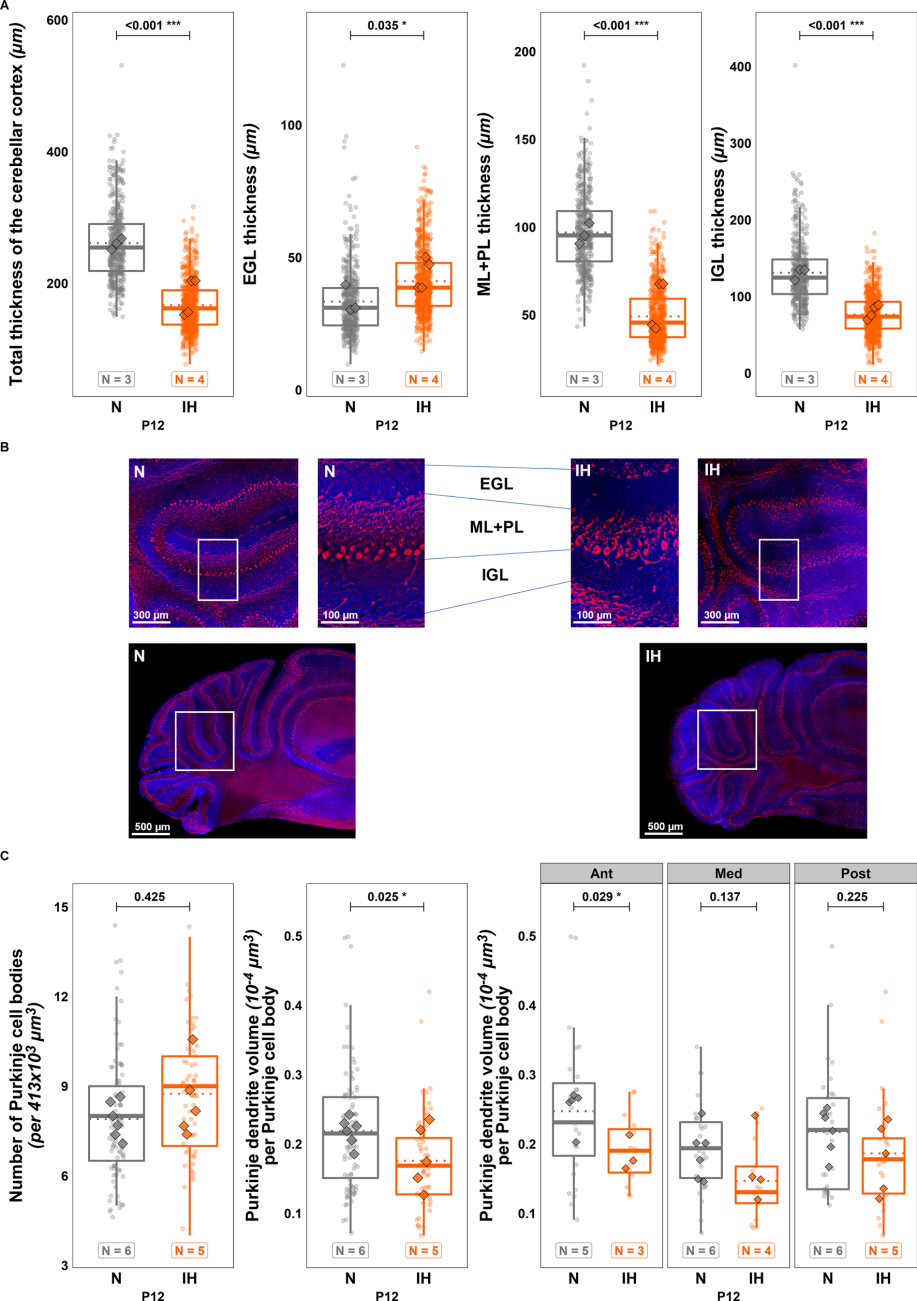
Effects of perinatal intermittent hypoxia on the organization of the developing cerebellum in P12 mice. **A** Measurement of the thickness of the cerebellar cortex and each cerebellar layer in control (N) or hypoxic (IH) P12 mice. **B** Low and high magnification confocal images illustrating the thickness of the cerebellar cortex layers in control (N) or hypoxic (IH) P12 mice. The Purkinje cells were labeled by antibodies against calbindin (red) and nuclei were counterstained with DAPI (blue). **C** Measurement of the number of Purkinje cell bodies per frame of 413 × 10^3^ µm^3^ (left) and of the volume of the calbindin-labeled Purkinje dendrites in the molecular layer of control (N) or hypoxic (IH) P12 mice, in the whole cerebellum (middle) and per cerebellar region (right). The total number of animals in each experimental group is indicated under the boxplots and represented by diamond shapes, while the transparent dots indicate individual data points. Exact p-values are indicated above the plot. EGL: external granular layer; IGL: internal granular layer; IH: intermittent hypoxia condition; N: normoxia condition; ML: molecular layer; P12: postnatal day 12; PL: Purkinje cell layer

At P12, immunofluorescent imaging revealed a disorganization of the cerebellar cortex in IH-treated mice compared to controls (Fig. 3A–C). The measurement of cerebellar cortex thickness at P12 showed a global reduction in IH mice ($t\left(1112\right)=4.099;p=<0.001;Ratio=1.503;C{I}_{95}=\left[1.237,1.827\right];$ Fig. 3A, B). This alteration is associated with a decrease in the IGL and ML thicknesses (respectively, $t\left(1112\right)=6.06;p=<0.001;Ratio=1.673;C{I}_{95}=\left[\mathrm{1.416,1.976}\right];$ and $t\left(1112\right)=3.789;p=<0.001;Ratio=1.781;C{I}_{95}=\left[\mathrm{1.321,2.402}\right];$ Fig. 3A, B) and an increase in EGL thickness ($t\left(1112\right)=-2.108;p=0.035;Ratio=0.816;C{I}_{95}=\left[\mathrm{0.676,0.986}\right];$ Fig. 3A, B), which further supports the delay in development. The immunochemical observation also revealed that the dendritic tree of Purkinje cells is visibly affected (Fig. 3B). The quantitative analysis of calbindin labeling confirmed the decrease of dendritic arborization volume per Purkinje cell body ($t\left(147\right)=2.26;p=0.025;Ratio=1.257;C{I}_{95}=\left[\mathrm{1.029,1.535}\right];$ Fig. 3C). The difference is more pronounced in the anterior part (Ant) of the cerebellum than in the medium (Med) or posterior (Post) part (Ant: $t\left(147\right)=2.209;p=0.029;Ratio=1.382;C{I}_{95}=\left[\mathrm{1.035,1.847}\right];$ Med: $t\left(147\right)=1.494;p=0.137;Ratio=1.235;C{I}_{95}=\left[\mathrm{0.934,1.632}\right];$ Post: $t\left(147\right)=1.219;p=0.225;Ratio=1.164;C{I}_{95}=\left[\mathrm{0.91,1.488}\right];$ Fig. 3C).

**Fig. 4:**
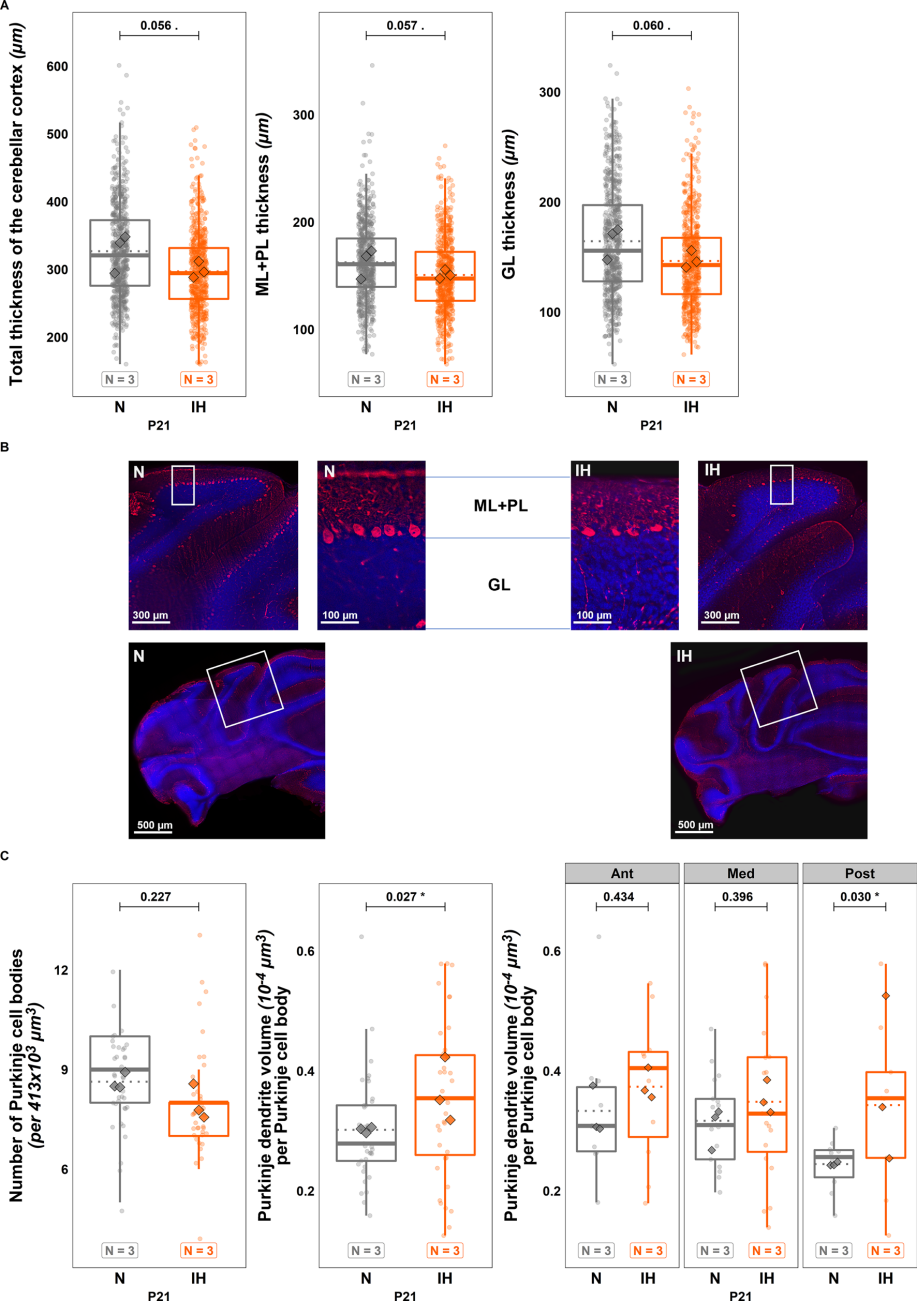
Effects of perinatal intermittent hypoxia on the organization of the developing cerebellum in P21 mice. **A** Measurement of the thickness of the cerebellar cortex and each cerebellar layer in control (N) or hypoxic (IH) P21 mice. **B** Low and high magnification confocal images illustrating the thickness of the cerebellar cortex layers in control (N) or hypoxic (IH) P21 mice. The Purkinje cells were labeled by antibodies against calbindin (red) and nuclei were counterstained with DAPI (blue). **C** Measurement of the number of Purkinje cell bodies per frame of 413 × 10^3^ µm^3^ (left) and of the volume of the calbindin-labeled Purkinje dendrites in the molecular layer of control (N) or hypoxic (IH) P21 mice, in the whole cerebellum (middle) and per cerebellar region (right). The total number of animals in each experimental group is indicated under the boxplots and represented by diamond shapes, while the transparent dots indicate individual data points. Exact p-values are indicated above the plot. GL: granular layer; IH: intermittent hypoxia condition; N: normoxia condition; ML: molecular layer; P21: postnatal day 21; PL: Purkinje cell layer

At P21, the thinning of the cortical layers is no longer significant (Fig. 4A, B). Moreover, the quantitative analysis of calbindin labeling demonstrated that the volume of the dendritic tree of Purkinje cells is increased in IH animals ($t\left(74\right)=-2.249;p=0.027;Ratio=0.832;C{I}_{95}=\left[\mathrm{0.708,0.979}\right];$ Fig. 4C). This effect is mostly accounted for by the posterior part of the cerebellum (Ant: $t\left(74\right)=-0.787;p=0.434;Ratio=0.891;C{I}_{95}=\left[\mathrm{0.666,1.192}\right];$ Med:$t\left(74\right)=-0.853;p=0.396;Ratio=0.908;C{I}_{95}=\left[\mathrm{0.724,1.138}\right];$ Post: $t\left(74\right)=-2.207;p=0.030;Ratio=0.713;C{I}_{95}=\left[\mathrm{0.525,0.968}\right];$ Fig. 4C). However, neither P12 nor P21 stages showed a significant difference in Purkinje cell count (Figs. 3C, 4C).

### Effects of perinatal intermittent hypoxia on physical and behavioral development

**Fig. 5:**
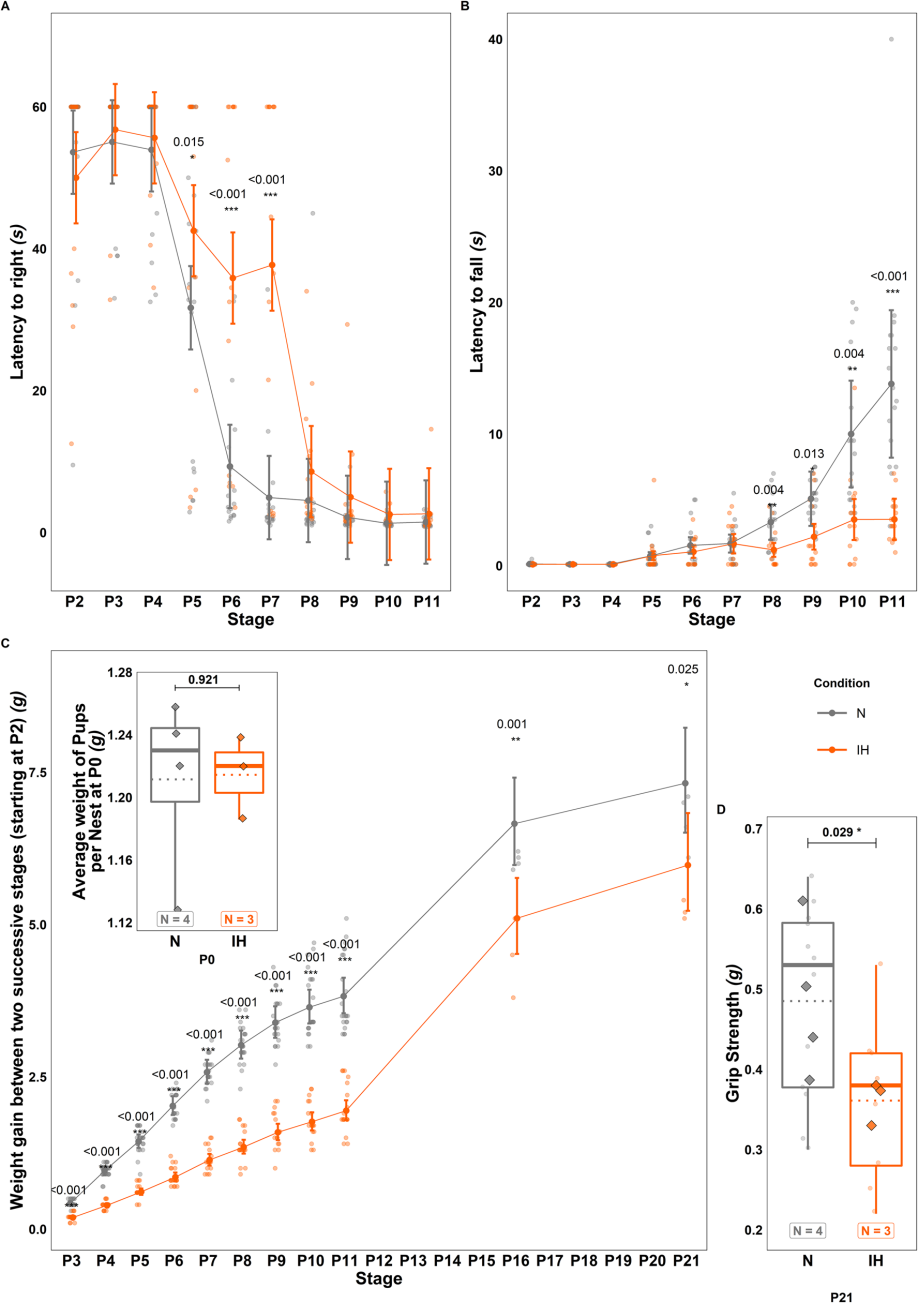
Effects of perinatal intermittent hypoxia on weight and behavioral development in young mice. **A**, **B** Measurement of the latency to turn in the righting reflex test (**A**) and to fall in the grasping reflex test (**B**) between P2 and P11 in 18 control (N) and 15 hypoxic (IH) mice. **C** Measurement of the weight gain of control (N) and hypoxic (IH) mice between P2 and P21 (N = 22, IH = 18 from P2 to P11—N = 4, IH = 3 from P12 to P21). Top-left insert: baseline control of P0 weight averaged from the total litter weight. **D** Measurement of the muscular strength using the forelimb grip strength test in control (N) and hypoxic (IH) P21 mice (3 technical replicates per animal). The total number of animals in each experimental group is indicated under the boxplots and represented by diamond shapes, while the transparent dots indicate individual data points. Exact p-values are indicated above the plot. IH: intermittent hypoxia condition; N: normoxia condition; Px: postnatal day x

**Fig. 6:**
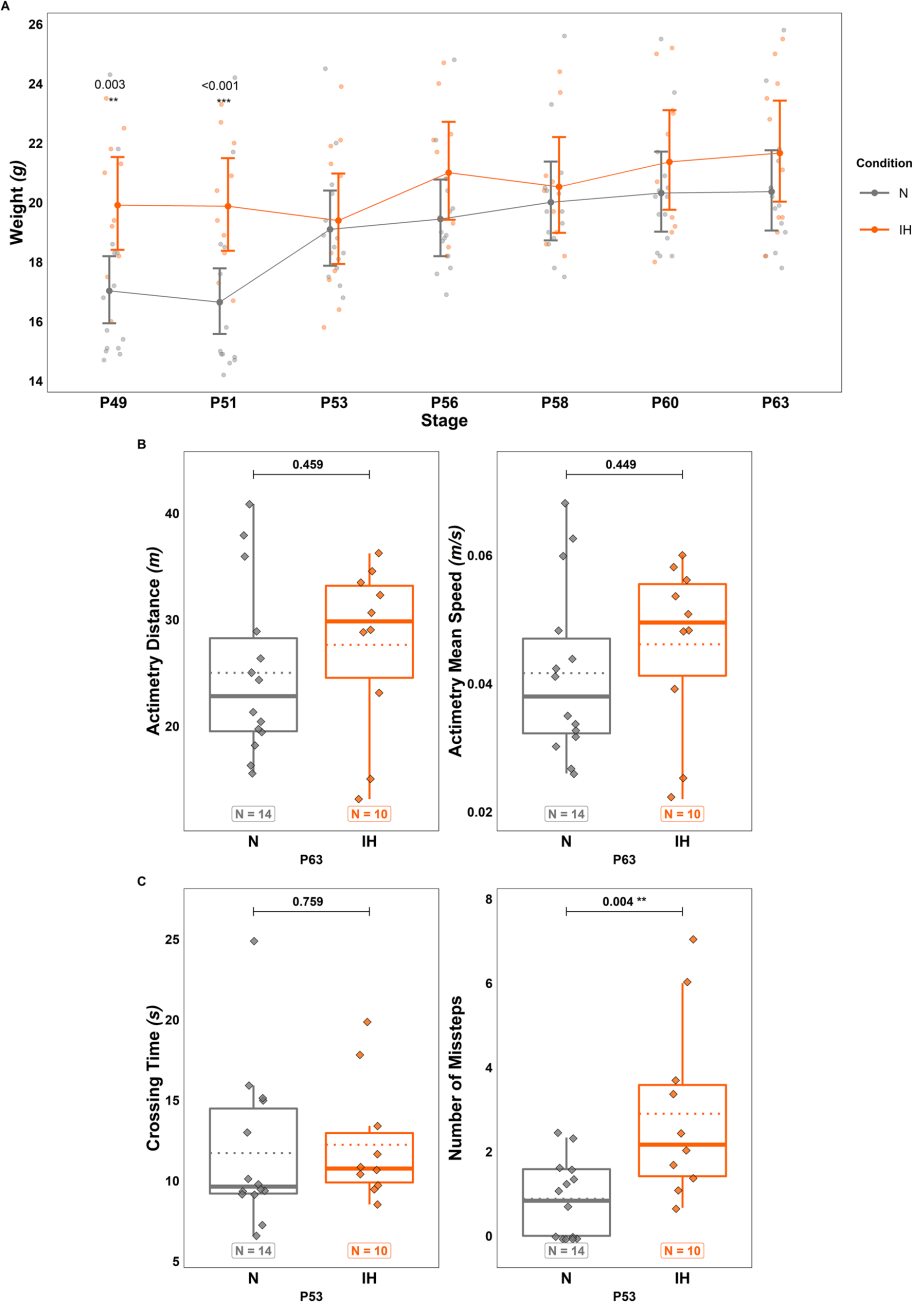
Long-term effects of perinatal intermittent hypoxia on locomotor activity and motor coordination in adult mice. **A** Measurement of the body weight in control (N) and hypoxic (IH) mice between P49 and P63. **B** Measurement of the distance travelled (left) and mean speed (right) in P63 control (N) and hypoxic (IH) mice during the actimetry test. **C** Measurement of the crossing time (left) and the number of missteps (right) in P53 control (N) and hypoxic (IH) mice during the horizontal beam test. The total number of animals in each experimental group is indicated under the boxplots in **B** and **C**, and exact p-values are indicated above. IH: intermittent hypoxia condition; N: normoxia condition; Px: postnatal day x

Two different tests were performed to determine whether perinatal IH could alter behavioral development in pups (N group = 11 males and 7 females; IH group = 7 males and 8 females). Firstly, we found that, in the righting reflex test, hypoxic mice take longer to turn at P6 and P7 compared to the control group (respectively + 26.56 ± 6.266 s and + 32.79 ± 6.45 s; $t\left(327\right)=-3.711;p=<0.001;Estimate=-7.931;C{I}_{95}=\left[-12.136,-3.727\right];$ Fig. 5A). Our data also revealed that, in the grasping reflex test, the hanging time of hypoxic mice is shorter than that of the control pups from P9 to P11 (respectively—2.92 ± 0.63 s; 6.52 ± 0.897 s;—10.29 ± 0.45 s;$t\left(327\right)=5.569;p=<0.001;Estimate=2.226;C{I}_{95}=\left[\mathrm{1.44,3.013}\right];$ Fig. 5B). Moreover, IH induces a growth delay in IH mice. Thus, while the mean weight of pups (nest weight/number of pups per nest) is similar at P0 ($t(3) = -0.107; p = 0.921; Estimate = -0.003; C{I}_{95} = [-0.091, 0.085];$ Fig. 5C, top left inset), animals display a lower daily weight gain from P2 to the end of the protocol ($t\left(331\right)= 13.971; p = <0.001; Ratio = 2.007; C{I}_{95} = [1.819, 2.213];$ Fig. 5C). No significant difference was found in weight gain by sex ($t(603) = 1.334; p = 0.183; Ratio = 1.048; C{I}_{95} = [0.978, 1.122];$ data not shown). This lower weight gain is associated with a significant effect on the muscular strength of mice at P21 ($t\left(19\right)=2.369;p=0.029;Ratio=1.34;C{I}_{95}=\left[\mathrm{1.035,1.735}\right];$ Fig. 5D). It even seems that an overcompensation occurs after weaning, since IH mice exhibit a significantly higher weight at P49 before stabilizing at the same level as controls at P53 (Fig. 6A).

### Long term effects of perinatal intermittent hypoxia on locomotor activity and motor coordination

To examine whether motor impairments due to perinatal IH in young mice could lead to long-term deficits in spontaneous locomotor activity and motor coordination, adult mice were subjected to actimetry and horizontal beam tests. Our results do not reveal any effect of IH on mean speed or on distance traveled (Fig. 6B). Similarly, IH animals spend the same time walking through the horizontal beam as control mice (Fig. 6C). However, hypoxic mice make more missteps on their path than controls at P53 (+ 229.17 ± 23.245%; $t\left(23\right)=-3.188;p=0.004;Estimate=-2.019;C{I}_{95}=\left[-3.329,-0.709\right];$ Fig. 6C).

### Long term effects of perinatal intermittent hypoxia on spatial learning

**Fig. 7:**
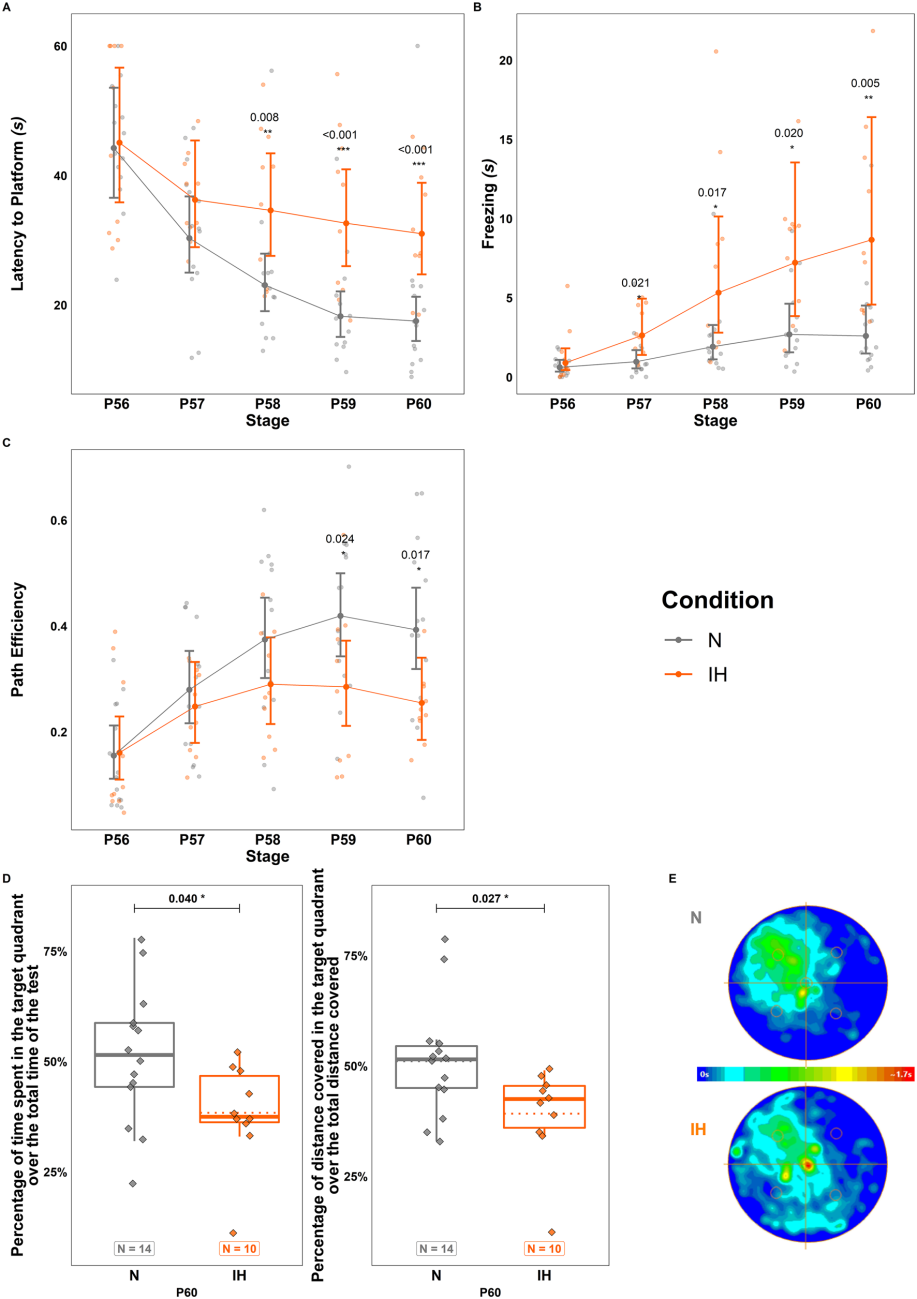
Long-term effects of perinatal intermittent hypoxia on spatial learning in adult mice. **A**–**C.** Measurement of the latency to find the platform (**A**), of the freezing time (**B**), and of the path efficiency (**C**) during the cue sessions of the Morris water-maze test performed in 14 control (N) and 10 hypoxic (IH) mice between P56 and P60. **D** Measurement of the time spent (left) and distance travelled (right) in the target quadrant during the probe session of the Morris water-maze test, performed on control (N) and hypoxic (IH) P60 mice. **E** Representative pattern of the time spent in the target quadrant during the probe session of the Morris water-maze test for one control mouse (N) and one hypoxic mouse (IH) at P60. Red and yellow regions represent areas of high occupancy; green and blue represent areas of low occupancy. The total number of animals in each experimental group is indicated under the boxplots in **D** and exact p-values are indicated above. IH: intermittent hypoxia condition; N: normoxia condition; Px: postnatal day x

As the cerebellum is now well known to participate in cognitive functions such as spatial navigation [20], we determined the impact of perinatal IH on adult mice performances in the Morris water maze test. We showed that IH mice are able to learn since their results improve over the 5 cue session days. However, they displayed an increased latency to find the platform and a prolonged freezing time (respectively, $t\left(117\right)=-3.607;p=<0.001;Ratio=0.704;C{I}_{95}=\left[\mathrm{0.581,0.854}\right];$ and $t\left(117\right)=-3.331;p=0.001;Ratio=0.399;C{I}_{95}=\left[\mathrm{0.232,0.689}\right];$ Fig. 7A, B), and presented an altered path efficiency ($t\left(117\right)=2.116;p=0.036;Odds.ratio=1.415;C{I}_{95}=\left[\mathrm{1.023,1.959}\right];$ Fig. 7C). Moreover, in the probe test, IH-treated mice spent less time (- 25.13 ± 9.51%; $t\left(23\right)=2.176;p=0.040;Odds.ratio=1.696;C{I}_{95}=\left[\mathrm{1.026,2.801}\right]$) and traveled a shorter distance in the platform quadrant (- 23.24 ± 8.712%; $t\left(23\right)=2.359;p=0.027;Odds.ratio=1.655;C{I}_{95}=\left[\mathrm{1.064,2.574}\right]$) compared to control mice (Fig. 7D, E).

### Long-term effects of perinatal intermittent hypoxia on anxiety

The effect of perinatal IH on anxiety was investigated in adult mice to check if the behavioral disorders observed in adults were due to distress. As shown in Additional file 2: Figure S1, the number of rearings and the grooming time, as well as the percentage of time spent in the center (data not shown) are similar in both groups of animals in the actimetry device. Moreover, in the elevated plus maze test, the time and number of entries in both closed and open arms are not significantly different for IH-treated and control mice (see Additional file 2: Figure S1). This indicates that IH mice do not display a more stressed or anxious behavior than the control animals.

### Long term effects of perinatal intermittent hypoxia on the functional organization of the cerebellum

To determine if the behavioral deficits observed in IH adult mice are due to a persistence of histological alterations in the cerebellum, we studied the structure of the cortical layers with a focus on Purkinje cells and their afferences.

**Fig. 8:**
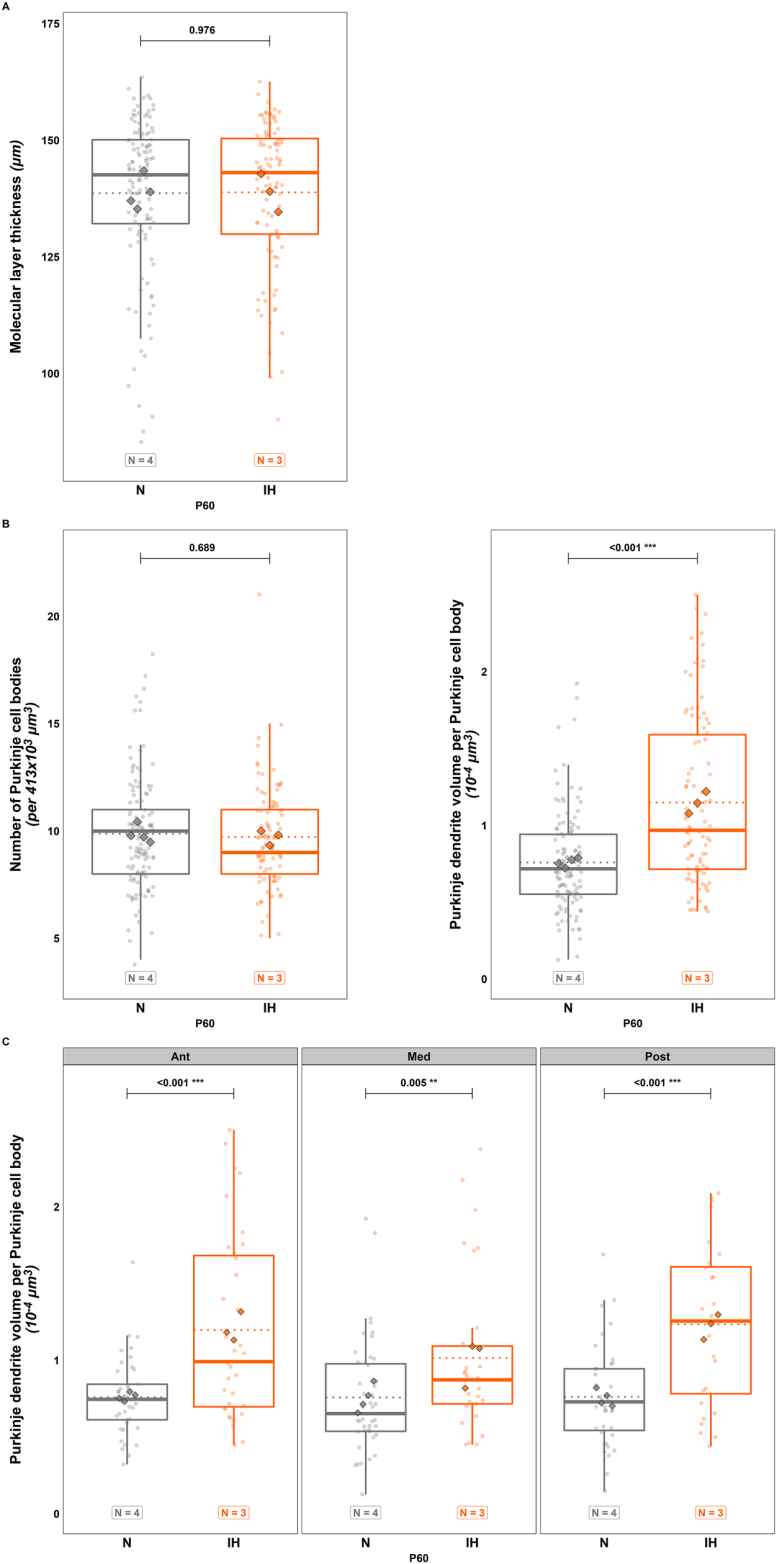
Effects of perinatal intermittent hypoxia on the structural organization of adult mouse cerebellum. **A** Measurement of the thickness of the molecular layer in control (N) and hypoxic (IH) P60 mice. **B**, **C.** Measurement of the number of Purkinje cell bodies (**B**, left) and of the volume of the calbindin-labeled Purkinje dendrites in the molecular layer of control (N) and hypoxic (IH) P60 mice, in the whole cerebellum (**B**, right) and per cerebellar region (**C**). The total number of animals in each experimental group is indicated under the boxplots and represented by diamond shapes, while the transparent dots indicate individual data points. Exact p-values are indicated above the plot. IH: intermittent hypoxia condition; N: normoxia condition; P60: postnatal day 60

Our results showed that there was no longer a difference in the thickness of the ML and in the Purkinje cell count between conditions in adulthood (Fig. 8A, B). In contrast, like at P21, the volume of Purkinje cell dendrites was significantly higher in the hypoxic group compared to the control animals ($t\left(215\right)=-6.795;p=<0.001;Ratio=0.663;C{I}_{95}=\left[0.5\mathrm{89,0.747}\right];$ Fig. 8B) and this can be observed in the entire cerebellum (Ant: $t\left(215\right)=-4.498;p=<0.001;Ratio=0.634;C{I}_{95}=\left[\mathrm{0.52,0.774}\right];$ Med: $t\left(215\right)=-2.826;p=0.005;Ratio=0.746;C{I}_{95}=\left[\mathrm{0.608,0.915}\right];$ Post: $t\left(215\right)=-4.438;p=<0.001;Ratio=0.617;C{I}_{95}=\left[\mathrm{0.498,0.764}\right];$ Fig. 8C).

**Fig. 9:**
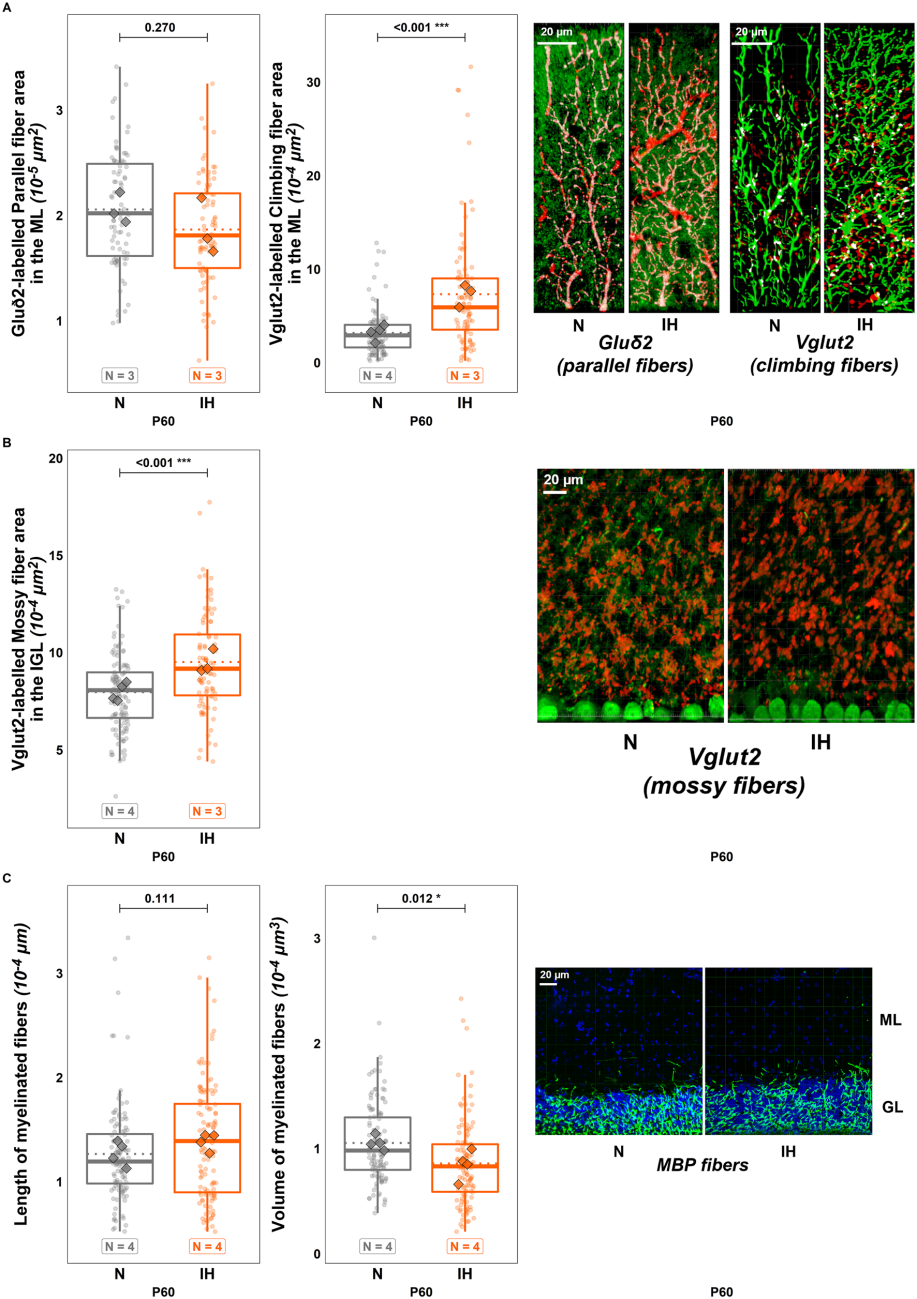
Long-term effects of perinatal intermittent hypoxia on the functional organization of adult mouse cerebellum. **A** Analysis of the Gluδ2-labeled parallel fiber area (left), and of the Vglut2-labeled climbing fiber area (middle) in the molecular layer of control (N) and hypoxic (IH) mice at P60. Measurements were done from confocal images as illustrated on the right. On Gluδ2 images, parallel fibers appear in green and calbindin-labeled Purkinje dendrites in red. On Vglut2 images, climbing fibers appear in red and calbindin-labeled Purkinje dendrites in green. The white labeling indicates the connection points between fibers and Purkinje dendrites. **B** Analysis of the Vglut2-labeled mossy fiber area in the granule cell layer (left) of control (N) and hypoxic (IH) mice at P60. Measurements were done from confocal images as illustrated on the right. Red labeling represents mossy fiber rosettes in the granule layer delimited by calbindin-labeled Purkinje cell bodies in green. **C** Measurement of the length (left) and the volume (right) of myelin binding protein (MBP)-labeled fibers in the granule cell layer in control (N) and hypoxic (IH) mice at P60. Measurements were done from confocal images as illustrated on the right. MBP-labeling (green) indicates that myelin sheaths are mainly located in the granule layer identified by a high DAPI-labeled nucleus density. Each box-plot represents the analysis of 2000 Purkinje cells from four different animals (Calbindin), 750 Purkinje cells from three different animals (Gluδ2), 1230 Purkinje cells from four different animals (Vglut2) and 120 confocal stack images of 10-µm thickness and 370-µm.^2^ surface from four different animals (MBP). The total number of animals in each experimental group is indicated under the boxplots and represented by diamond shapes, while the transparent dots indicate individual data points. Exact p-values are indicated above the plot. IH: intermittent hypoxia condition; N: normoxia condition; P60: postnatal day 60

In parallel, a double staining with calbindin and glutamate receptor delta2 (Gluδ2) or vesicular glutamate transporter 2 (Vglut2) was performed to analyze the connections between the Purkinje cell dendrites and parallel or climbing fibers, respectively. Although we did not see any difference in Gluδ2 labeling (Fig. 9A), our results show an increase of Vglut2-labeled fibers in IH mice ($t\left(207\right)=-4.507;p=<0.001;Ratio=0.431;C{I}_{95}=\left[\mathrm{0.298,0.623}\right];$ Fig. 9A). Vglut2 labeling, which is specific to mossy fibers in the adult granule cell layer, enabled us to demonstrate that IH increases the density of mossy afferences in adult animals ($t\left(208\right)=-4.448;p=<0.001;Ratio=0.837;C{I}_{95}=\left[\mathrm{0.774,0.906}\right];$ Fig. 9B).

Since hypomyelination is one of the major alteration observable in humans, we performed a staining for MBP and analyzed labeled fibers in the IGL. Interestingly, the difference in fiber length was not significant (Fig. 9C) but a significantly lower fiber volume was found in the hypoxic group ($t\left(238\right)=2.529;p=0.012;Ratio=1.249;C{I}_{95}=\left[\mathrm{1.05,1.485}\right];$ Fig. 9C).

## Discussion

The development of the cerebellum begins during the embryonic stage but continues well into the postnatal period [12]. Indeed, this structure is immature at birth and thus particularly vulnerable to several perinatal incidents. As a result, it has been shown that it is affected in premature newborns who suffer, among other things, from cerebellar atrophy [21, 22]. However, the consequences of apnea of prematurity, a common pathology affecting over 50% of premature newborns, have not been studied on the cerebellum, even though the behavioral deficits observed in children suffering from this pathology are correlated with cerebellar functions [6, 7]. Based on these data, we undertook to investigate the effects of IH on cerebellar development in a murine model of AOP based on the works of Cai et al. [8]. While our protocol does not induce any mortality, we showed that it leads to growth retardation in mice. The effect of IH on the body weight of pups is well known [23–25]. It has been demonstrated that it is not due to an impact on the mothers, since perinatal IH causes a weight decrease in pups, regardless of the presence of hypoxic dams in the litter [23]. Moreover, we did not observe any weight difference, stress or behavioral changes in mothers during our hypoxia protocol (data not shown). We cannot exclude that IH may cause a lower quality in the dams’ milk production or coordination problems affecting pup suckling during lactation, although no data have been published about this hypothesis in the literature. Moreover, the fact that the difference in weight gain tended to decrease between IH and N animals from P16 onwards is in favour of IH-related fatigue or general stress, inducing a decrease in milk intake, which would then fade at the end of the protocol.

Concerning neurodevelopment, perinatal hypoxia is known to affect various brain regions such as the corpus callosum, striatum, frontal cortex, hippocampus, and cerebellum [11, 26, 27]. We confirm here that the cerebellum is a major target of perinatal IH since we observed signs of oxidative stress in this structure at P12.

Indeed, our IH protocol induces an overproduction of ROS supported by the overexpression of the genes Hmox1, Idh1 and Hif1α, known to be sensors of oxidative stress [28–30]. We also observed a decrease of Cox4i1 and Fth1 expression, which could contribute to ROS accumulation [31, 32]. Similarly, several antioxidant enzymes are less expressed at P12, such as the glutathione peroxidases, the superoxide dismutase 1, the peroxiredoxin 1, the sulfiredoxin 1 and the glutathione-S-transferase kappa 1 [33–36], indicating that cerebellar cells have suffered from the hypoxia. This oxidative stress is accompanied by an overall decrease in the thickness of the cerebellum that affects all cortical layers. This thinning could be due to an increase of apoptosis as qPCR experiments show that the genes for numerous pro-apoptotic enzymes, including caspases 3 and 9 are upregulated and the anti-apoptotic factor Bcl2 is under-expressed after IH at P12. However, we do not see any changes in cleaved caspase-3/7 activity and in the number of cleaved caspase-3 immunoreactive cells, suggesting that apoptosis is not or no longer exacerbated at the end of the protocol. One explanation for this discrepancy could be the establishment of a protective system against oxidative stress. Indeed, IH decreases the expression of the pro-oxidant genes Ndufv2 and Nos1 [33, 37], while significantly increasing the expression of antioxidant enzymes’ genes for superoxide dismutases 2 and 3, glutathione-S-transferase mu and thioredoxin reductase 3 [36, 38, 39]. This compensatory mechanism could balance the increased apoptotic cell death as suggested by the decrease in Parp1 expression [19], leading to the same cell density in all cerebellar layers at the end of the IH protocol. Therefore, the thickness decrease could instead be imputable to a general delay in cerebellar maturation consistent with the global growth retardation. This hypothesis is supported by our immunohistochemical analysis showing that, in IH mice, the proportional thickness of each cortical layer is close to that of a P6 cerebellum with a thicker EGL and thinner ML and IGL. We also revealed that hypoxia increases the number of BrDU-positive cells in the EGL, indicating that IH maintains a high proliferative activity of GCP at P12 much like that of an immature cerebellum. In contrast, no difference in the number of BrDU-positive cells was seen in the IGL. This result shows that precursors that proliferated between P6 and P12 did not have difficulty reaching the IGL despite of IH, suggesting that hypoxia does not affect GCP migration. Moreover, IH does not modify the cell density in the ML and IGL, which respectively contain stellate and basket cells; and granule, Golgi and Lugaro interneurons, suggesting that these neurons are not (or no longer) significantly affected by IH at P12. The weak impact of IH on granule cells is confirmed by our Gluδ2 immunohistochemical analysis in adult mice, which shows no difference in the parallel fiber innervation of Purkinje cells.

However, by focusing on Purkinje cells, we determined that, while the number of cell bodies is equivalent in both groups, the volume of their dendritic tree is lower at P12 post IH. These data revealed that hypoxia induces a delay in Purkinje cell differentiation that mainly occurs in the anterior part of the cerebellum. This differential sensibility to hypoxia has already been shown in rodents as well as in humans and demonstrates that the anterior part of the cerebellum is usually more affected by a perinatal deprivation of oxygen [40, 41]. It could be explained by the developmental timing of the cerebellum, which begins with lobules I-III and ends with the separation of lobules IV-V [42, 43]. Thus, the posterior regions were already mature at P2 when we started the IH protocol, while the foliation of the anterior part was still ongoing until P5. Similarly, it has been demonstrated that Purkinje cells located in the anterior cerebellum become mature later than in the posterior region [43], suggesting that this region-specific immaturity could make Purkinje cells more sensitive to perinatal hypoxia.

Since Purkinje cells represent the cerebellum’s information integration center, we performed behavioral tests to determine if IH could lead to functional deficits in addition to structural alterations. Indeed, between P2 and P12, IH-treated pups show locomotor deficits, such as a delayed righting reflex and diminished grasping reflex. While these differences could be due, in part, to a gross morphological underdevelopment and lower weight, they are also consistent with a dysfunction of the anterior part of the cerebellum known to be implicated in sensori-motor control [44].

At P21, although mice still display a significantly lower body weight and reduced muscular strength, the difference in the histological organization of the cortical layers is no longer observed, indicating that a compensatory mechanism occurred since the end of the IH protocol. This process was also effective on Purkinje cells since these neurons no longer present a decrease of their dendritic tree volume. Indeed, they exhibit a denser arborization that mainly appears in the posterior part of the cerebellum. This result suggests that the compensatory mechanism takes place throughout the cerebellum and allows the IH neurons to catch up with the cells of control animals in the anterior and medium parts. However, this compensation induces an overdevelopment of the Purkinje dendritic tree in the posterior region, which was not altered by hypoxia at P12. These data led us to study the long-term effects of perinatal IH in adulthood.

Starting at P49, the body weight of IH mice caught up with and even transiently surpassed the controls. Likewise, the ML is similar in both conditions. However, we showed that functional impairments, such as the motor coordination deficits assessed by the horizontal beam test, are still present in adult mice that have experienced perinatal hypoxia. Moreover, these animals have lower performances in both cue and probe sessions of the Morris water-maze test. The increased freezing time could suggest a potentiated stress behavior but the results obtained in the actimetry and elevated plus maze tests do not reveal any anxiety in IH-treated mice (see Additional file 2). Thus, our results indicate that perinatal IH induces long-term motor function deficits associated with learning and/or even orientation impairments in adulthood. Such a deficit in spatial memory after hypoxia has already been demonstrated but it has been mainly attributed to decreased synaptic plasticity in hippocampal neurons [10]. However, the role of the interaction between cerebellum and hippocampus in the control of spatial navigation is now well known [45, 46], indicating that the perinatal IH-induced alteration of the cerebellum could also be responsible for this behavioral deficit.

Since, in the hippocampus, IH decreases synaptic plasticity and long-term potentiation [10], we examined the morphology of Purkinje cells and studied cerebellar afferences. We found that, in adult IH mice, Purkinje dendrites are denser, not only in the posterior part as in P21, but throughout the cerebellum. Thus, the locomotor and spatial learning deficits could be linked to the alteration of the anterior and the posterior parts of the cerebellum, respectively [44, 47, 48].

Concerning afferences, a high Vglut2 labeling was detected in the ML of IH mice, suggesting that cerebellar innervation by climbing fibers is also affected by hypoxia. During development, Purkinje neurons receive inputs from multiple climbing fibers, but they are progressively eliminated during the first two postnatal weeks and only one afference per Purkinje cell will persist in adulthood [49]. Two selection steps occur in physiological conditions: a granule cell-independent phase between P6 and P12 and a later phase, between P12 and P17, which relies on the activity of parallel fibers [50]. Since our IH protocol was applied before P12, we can hypothesize that hypoxia mostly altered the early selection phase leading to the over-innervation of Purkinje cells by climbing fibers. This hypothesis is further reinforced by the Gluδ2 immunolabeling that showed no difference in the parallel fiber innervation of Purkinje dendrites.

Similarly, an increase in Vglut2 labeling was seen in the IGL, indicating that mossy fibers may also be impacted by perinatal IH. These afferences originate from various central nervous regions, such as the spinal cord or brainstem nuclei, enter the cerebellum at birth and make contacts with granule cells in the mature cerebellum. Several studies have shown that granule neuron activity is important for an appropriate innervation during the first postnatal week [50], and we found here that the marker of Purkinje cell-parallel fiber synapses Gluδ2 reveals no difference in adult mice. However, it is known that mossy fibers first connect Purkinje cells before moving on to mature granule cells as the IGL appears [50, 51]. Therefore, we can hypothesize that the delay in IGL formation after hypoxia allows a high late mossy fiber innervation that persists in adulthood. In fact, this differential mossy input according to the maturation degree of granule cells has already been suggested [52, 53]. Alternatively, the reduction of the Purkinje dendritic tree density at P12 in IH mice could also increase the switch to contact with granule cells and later could induce an over-innervation in adulthood.

Finally, we show that the volume (but not the length) of myelin sheaths is decreased in adult mice after a perinatal IH, which is consistent with numerous studies [10, 11, 54]. However, although most of these works analyzed cerebellar white matter, we focused on the granule cell layer. Since mossy and climbing fibers mostly lose their myelin sheaths when entering the cerebellar cortex, our results demonstrate that the myelination defect mainly concerned Purkinje axons.

## Conclusion

We have investigated the effects of a model of apnea of prematurity on cerebellar development in mice. Here, we demonstrated that perinatal IH induces modifications of cellular mechanisms such as oxidative stress, leading to a delay in cerebellar cortex maturation. This developmental retardation seems to be compensated by mechanisms such as an increased proliferation, allowing the cerebellar cortex to achieve its development. However, some structural modifications, including denser dendritic arborization of Purkinje cells, over-innervation by cortical afferences and axon hypomyelination, persist after IH and induce short and long-term impairments affecting motor coordination, learning and/or orientation. Such a link between Purkinje cell development failure, afferent innervation and long-term behavioral deficits has already been proven in another pathological context [55] and suggests that apnea of prematurity can lead to structural and functional alterations within the cerebellum, that may participate, at least in part, in the observed neurodevelopmental disorders.

## Methods

### Animals

Animals used in this study were wild type C57/Bl6J mice born and bred in an accredited animal facility (approval B.76–451-04) in accordance with the French Ministry of Agriculture and the European Community Council Directive 2010/63/UE of September 22^nd^, 2010, on the protection of animals used for scientific purposes. The mice were kept under a 12-h light/dark cycle and had free access to food and water. Due to the age of mice at the beginning of the hypoxia protocol (P2), it was not feasible to manage the sex ratio. However, the sex of animals was determined at P12 at the end of the protocol. For immunolabeling, enzyme activity and behavioral tests, 18 N (11 males-7 females) and 15 IH (12 males-3 females) pups have been used at P12, 4 N (3 males-1 female) and 3 IH (2 males-1 female) juvenile animals at P21, and 14 N (5 males-9 females) and 10 IH (6 males-4 females) mice in adulthood. For qRT-PCR, 15 N (7 males-8 females) and 10 IH (7 males-3 females) have been used at P12. For all experiments, no sex dimorphism was observed (data not shown). Each animal was weighed at 9:00 each day before the beginning of the IH protocol. To avoid handling animals individually before P2 because of the stress-induced cannibalism of the mother, the whole nests were weighed at P0 when the pups were counted. Then, from P2, animals were identified and their weight gain was measured individually until P12 (or P21). There was no blinding in this study and sample size determination was done empirically based on bibliographic data.

### Intermittent hypoxia protocol

The model of IH is based on the paradigm developed by Cai et al. [8] and consisted of 2-min cycles of hypoxia (5% O_2_; 20 s/cycle) and reoxygenation for 6 h per day. The protocol was initiated in neonatal P2 C57Bl6/J pups (IH group) and continued for 10 consecutive days. Oxygen concentration, temperature, hygrometry, and atmospheric pressure in the hypoxic chamber were continuously monitored. Control normoxia pups (N group) were placed in another chamber in the same environment but in normoxic conditions. Upon completion of an IH course, some mice were used for enzymatic and oxidative assays. For histological studies, P12 or P21 mice were lethally anesthetized by intraperitoneal injection of ketamine (100 mg/kg) and xylazine (10 mg/kg) and then sacrificed by intracardiac perfusion of NaCl 9‰ and paraformaldehyde 4%. Brains were rapidly removed, fixed overnight in 4% paraformaldehyde and then stored in phosphate buffer saline (PBS) prior to section. The remaining mice were raised until P64 for behavioral assessments.

### Reactive oxygen species (ROS) production assay

100 µL of homogenized cerebellum were incubated with 2 µL of 2’,7’-dichlorofluorescein diacetate (Sigma-Aldrich). In the presence of ROS, this probe is converted to fluorescent 2’,7’-dichlorofluorescein. The fluorescence intensity was measured at an excitation wavelength of 485 nm and an emission wavelength of 530 nm every 15 min over 45 min with an Infinite 200 microplate reader (Tecan). Data were standardized after protein quantification of each sample with a Bradford assay.

### Expression of genes involved in oxidative stress and apoptosis

#### Sample gathering

Mice were sacrificed by decapitation after completion of the IH protocol at P12. Cerebella were immediately harvested and set into isopentane kept at -30 °C. They were then stored in sterile containers at -80 °C until further use.

#### Primer design

Gene primers were designed with the software Primer Express (v3.0.1; ThermoFischer Scientific) using nucleotide sequences from the NCBI Pubmed database. They were classified into 3 panels according to the functional enrichment in the GO database: ROS production (9 genes), antioxidant response (23 genes) and apoptosis (6 genes). Primer pairs were ordered from Integrated DNA Technologies and validated by linear regression of serial dilution data. The list of primer pair sequences is available in Additional file 3: Table S2.

#### RNA Extraction

The mRNAs were purified on column using the Nucleospin RNA extract II extraction kit by Macherey–Nagel (cat.740955250) according to manufacturer recommendations. mRNA concentration and purity were controlled by UV spectrophotometry on the NanoDrop One (ThermoFisher Scientific) and quality assessment was performed by gel electrophoresis on RNA 6000 Pico chips (cat. 5067–1513, Agilent). The mRNAs were then stored at −80 °C until the next step.

#### qRT-PCR

The determination of the expression level of genes was done by quantitative PCR in 384-well plates with a 5µL reaction volume in the presence of Fast SYBR Green PCR Mastermix (Thermofisher, cat. 4,385,612). The distribution of cDNA samples and reaction mixes was performed by the Bravo 1 Automated Liquid Handling Platform (Agilent). The quantitative PCR reaction took place in the QuantStudio Flex 12 k thermal cycler (Applied Biosystems). Two technical replicates per animal and per gene of interest (GOI) were averaged prior to DCq calculation. The average Cq value of three housekeeping genes (HKG) was used to calculate DCq. Efficiency for all primer pairs was above 95% and as such, the 2^(−ΔΔCq)^ method can be applied confidently. This method provides a relative quantification of the expression of a gene within an experimental condition and makes it possible to to compute fold change for the graphic representation. This value is defined by the formula

$${2}^{-\Delta \Delta Cq}={2}^{\big(-\left(\left({Cq}_{GOI IH}-{Cq}_{HKG IH}\right)-\left({Cq}_{GOI N}-{Cq}_{HKG N}\right)\right)\big)}$$

### Caspases-3/7 activity assay

The enzymatic activity of caspases-3/7 in the cerebellum was evaluated with the Apo-ONE® Homogeneous Caspase-3/7 Assay (Promega). 100 µL of homogenized cerebellum were incubated at 37 °C with 100 µL of caspase assay buffer containing the Z-DEVD-aminoluciferin substrate. The fluorescence intensity was measured at an excitation wavelength of 485 nm and an emission wavelength of 530 nm every 6 min over 3 h with an Infinite 200 microplate reader. Data were standardized after protein quantification of each sample with a Bradford assay.

### Bromodeoxyuridine (BrDU) injections

Animals received intraperitoneal administration of BrdU (50 mg/kg) at P6 to assess the capacity of GCP to divide and migrate, and then, at P12, 4 h before euthanasia, to analyze immediate proliferation of GCP.

### Immunolabeling

For immunohistochemical studies, fixed brains were cut into frontal 80-µm thick slices with a vibratome (VT1000S, Leica Microsystems). Slices were then blocked 30–60 min at room temperature with normal donkey serum diluted at 1:50 in a buffer containing 0.3% Triton X-100. Then, the slices were incubated at 4 °C with primary antibodies diluted in the same buffer (Table 1). For BrDU labeling, a step of DNA denaturation consisting in a 15-min incubation in an HCl 1 N solution at 45 °C was done before adding BrDU antibodies. After three 10-min washes in PBS, they were incubated at room temperature for 2 h with the corresponding secondary antibodies diluted at 1:250 (Table 1). The slices were rinsed three times for 10 min in PBS and labeled for 1 min with 4′,6-diamidino-2-phenylindole (DAPI; 2 µg/mL) for nucleus counterstaining before the last wash in PBS. Finally, they were mounted with Mowiol before image acquisition.

**Table 1:** Summary table of immunohistochemical markers used in this study

Primary antibody	Marker	Dilution	Species	Provider	Secondary antibody	Incubation time
BrDU	Proliferating cells	1/400	Sheep	Abcam (#ab1893)	DAS-633	O/N at 4 °C
Calbindin	Purkinje cells	1/1000	Mouse	Sigma-Aldrich (#C9848)	DAM-594	O/N (or 72 h with Gluδ2 and Vglut2) at 4 °C
Cleaved-caspase-3	Apoptotic cells	1/400	Rabbit	Cell Signaling Technology (#9661S)	DAR-488	O/N at 4 °C
Gluδ2	Parallel fibers	1/500	Goat	Santa Cruz (#sc26118)	DAG-488	72 h at 4 °C
MBP	Myelinated fibers	1/300	Rabbit	Sigma-Aldrich (#M3821)	DAR-488	O/N at 4 °C
Vglut2	Climbing fibers + Mossy fibers	1/500	Guinea pig	Millipore (#AB2251)	DAGp-Cy3	72 h at 4 °C

*BrdU* bromodeoxyuridine, *DAG* donkey anti-goat, *DAGp* donkey anti-guinea pig, *DAM* donkey anti-mouse, *DAR* donkey anti-rabbit, *DAS* donkey anti-sheep, *Gluδ2* glutamate receptor delta2, *MBP* Myelin binding protein, *O/*N overnight, *Vglut2*: vesicular glutamate transporter 2

### Image analysis

Frontal sections were chosen to acquire symmetric images from as many lobules as possible on a minimum of slices and thus obtain a large panel of data. For each animal, 8 slices were harvested in the whole cerebellum and assigned to one of three groups according to Bregma coordinates, namely, the posterior (from −8.5 to −7.5 mm), the medium (from −7.5 to −7.0 mm) and the anterior parts (from −7.0 to −6.0 mm) (see Additional file 4).

For the measurement of layer thickness, image acquisition was done with a conventional Eclipse 600D microscope (Nikon) using a × 20 objective. Mosaics were manually reconstructed prior to whole-slice measurements on the Fiji software [56, 57].

For the cell density measurement and the counting of BrDU and caspase-3-positive cells, images were acquired with a confocal microscope (TCS SP8 MP, Leica Microsystems). Each image corresponds to a 20-μm thick section with 4-μm step z-stacks (2048 × 2048 focal planes) acquired using a × 20 objective. Analysis was done on the Imaris software (Bitplane, Zurich, Switzerland) and all three-dimensional reconstructions were done with the same threshold.

For the study of calbindin, Gluδ2, Vglut2 and myelin binding protein (MBP) immunolabeling, image acquisition was completed on a confocal microscope (TCS SP8 MP, Leica Microsystems). Each image corresponds to a 10-μm thick section with 1-μm step z-stacks (2048 × 2048 focal planes) acquired using a × 40 objective. Analysis was done using the Imaris software (Bitplane, Zurich, Switzerland) and all three-dimensional reconstructions were done with the same threshold.

### Behavioral studies

Behavioral assessments were performed in blind condition between 10:00 a.m. and 5:00 p.m. At least one hour before the beginning of each test, animals were familiarized with the testing room. The apparatus were cleaned with an alcohol solution (10% ethanol) and dried before testing each animal.

#### Righting reflex test

This test evaluates the activity of the vestibular, articular, and muscular structures as well as movement completion. Pups were placed in a supine position and the time needed to recover a completely prone position was measured. Two daily consecutive trials were performed on each pup from P2 to P11 with a 60-s cut-off period.

#### Grasping reflex test

This experiment appraises the animal's grasping reflex and motor coordination. Pups were hanged by their forepaws on a stretched string and the latency to fall was measured. Two daily consecutive trials were performed for each pup from P2 to P11 with a 60-s cut-off period.

#### Muscular strength test

This test, which monitors the muscle strength of animals, can only be performed from P21 onwards***.*** Mice were held by the tail and allowed to grasp a string with their forepaws. Then, they were progressively pulled backward by the tail. The maximal force was recorded by a dynamometer linked to the grip-strength apparatus. Three trials were performed on each mouse at P21.

#### Actimetry test

This test evaluates the spontaneous locomotor activity of animals in a new environment. In this session, P63 mice were placed in the middle of a 45 × 45 × 30 cm box, and their spontaneous locomotor activity was recorded for 10 min with the video tracking software Any-MAZE. Various parameters, such as the distance traveled and mean speed were measured, and the grooming time as well as the number of rearings were logged (see Additional file 2).

#### Elevated plus maze

This test estimates the anxiety state of the animals. P64 mice were placed at the intersection of a maze consisting of 2 opposed closed arms crossing 2 opposed open arms. The movements of the animals were tracked for 5 min with the Any-MAZE software. The time spent and the number of entries in each arm were recorded (see Additional file 2).

#### Horizontal beam test

This test assesses anxious behavior in mice. Mice were placed on a wooden beam (1 m in length, 0.5 cm in diameter), and the time needed to cross the beam was measured, as well as the number of missteps. Three trials were performed for each mouse at P53.

#### Morris water-maze test

This test studies spatial learning and memory. It requires a round pool of 120 cm in diameter, surrounded with visual clues and filled with water mixed with an opaque white dye. Each mouse aged P53 was given 60 s to habituate to the pool. Then, a cue session was performed at P56 and during 5 consecutive days, corresponding to the acquisition and consolidation phases. An invisible platform was placed in the northwest quadrant and submerged approximately 1 cm below the water surface. Each animal was placed in the pool and had 60 s to find the platform and to stay on it for 5 s. If mice did not find the platform, they were gently guided to it and stayed there for 20 s. Four trials with different starting positions were performed each day. On the last day, a probe session was conducted, referring to the retrieval phase. The platform was removed from the pool and each mouse was placed in the center of the pool. Mice were given 60 s to explore the environment. Their behavior and different parameters such as the freezing time or distance traveled were analyzed with the video tracking software Any-MAZE.

### Statistical analysis

Statistical analyses were performed within the R statistical computing environment (version 4.1.3) [58]. Model fitting was done using the "glmmTMB" package [59], and model diagnostics were done using the "DHARMa" [60] & "performance" [61] packages. Expected marginal means and contrasts were computed with the "emmeans" package [62]. P-values for the relevant contrasts were computed on the link scale, using Wald t-tests, without any adjustments. For all analyses, p < 0.05 was considered significant.

Data from image acquisition following immunolabeling were modeled within the Generalized Linear Mixed Model (GLMM) framework, with a random intercept per mouse to account for the correlation between pseudo-replicates taken from the same mouse [63]. Temporal data (i.e. weight changes, righting and gripping reflexes, and Morris water maze) was analyzed within the Generalized Linear Model (GLM) framework, using an autoregressive factor of order one (AR1) to account for the dependencies between the repeated measures.

For RT-qPCR data model fitting and subsequent statistical testing was done on DCq values. We used a linear mixed effect model with a random effect by plate to account for fluorescence variability between experiments.

For each response variable, the optimal likelihood families were selected based on our theoretical understanding of each variable’s properties and based on Aikake's Information Criterion (AIC). Count data (e.g., cell counts, number of maze entries, …) were modeled using a Generalized Poisson likelihood family, measures bound at 0 (e.g., cell density, volumes, weights, …) were modeled using a Gamma likelihood, and proportions (e.g., ratios of areas) with a Beta likelihood family. The only exception was the righting reflex measures, which were modeled using a Gaussian due to the censored nature of the data resulting in unrealistic expected values with other more theoretically appropriate distributional families.

## Supplementary Information

**Additional file 1: Table S1.** Full statistical summary of RT-qPCR data.

**Additional file 2: Figure S1.** Long-term effects of potential intermittent hypoxia on anxiety in adult mice.

**Additional file 3: Table S2. **List of primers used for RT-qPCR experiments and their corresponding sequences.

**Additional file 4: Figure S2.** Cerebellar frontal illustrations indicating the allocation of the slices used for immunuhistochemical analyses and their assignment to one of three groups according to Bregma coordinates.

## Data Availability

The datasets supporting the conclusions of this article are available in the following GitHub repository at https://github.com/ma-riviere/LT-AoP-22, also referenced on Zenodo under: https://doi.org/10.5281/zenodo.6480947.

## Abbreviations

AOP: Apnea of prematurity
Ant: Anterior part of the cerebellum
BrDU: Bromodeoxyuridine
EGL: External granular layer
GCP: Granular cell precursors
Gluδ2: Glutamate receptor delta2
IGL: Internal granular layer
IH: Intermittent hypoxia
MBP: Myelin binding protein
Med: Medium part of the cerebellum
ML: Molecular layer
N: Normoxia
PL: Purkinje cell layer
Px: Postnatal day x
Post: Posterior part of the cerebellum
ROS: Reactive oxygen species
VGlut2: Vesicular glutamate transporter 2
