# Supplementary material for: Intermittent hypoxia in a mouse model of apnea of prematurity leads to a retardation of cerebellar development and long-term functional deficits
Source: Cell Biosci. 2022 Sep 6;12:148. doi: 10.1186/s13578-022-00869-5 (PMC9450451; doi:10.1186/s13578-022-00869-5)
Supplement: Supplementary file 1 — Additional file 1: Table S1. Full statistical summary of RT-qPCR data. [file 13578_2022_869_MOESM1_ESM.docx]

| Gene | Fold Change | Mean DCq N | Mean DCq IH | Difference | CI 95 | t | Hedge g | p Value |
| --- | --- | --- | --- | --- | --- | --- | --- | --- |
| Bax | 1.073375864 | 5.054340496 | 5.035602139 | 0.088264285 | [-0.049, 0.23] | 1.34069274 | 0.091756692 | 0.194 |
| Bcl2 | **0.813401016** | **6.905556367** | **7.186905812** | **-0.28967436** | **[-0.37, -0.21]** | **-7.81876947** | **-2.597825303** | **<0.001 ***** |
| Casp3 | **1.227772125** | **5.160688025** | **4.870444249** | **0.290338752** | **[0.18, 0.4]** | **5.65278443** | **2.012341332** | **<0.001 ***** |
| Casp9 | **1.19077743** | **8.567307924** | **8.349436329** | **0.248081341** | **[0.13, 0.37]** | **4.31806231** | **0.769238237** | **<0.001 ***** |
| Cat | 0.956480377 | 5.003649146 | 5.113754033 | -0.076221338 | [-0.17, 0.017] | -1.69895065 | -0.859118513 | 0.104 |
| Cox4i1 | **0.918959058** | **1.221241639** | **1.356367444** | **-0.135449162** | **[-0.26, -0.008]** | **-2.20944076** | **-0.838161711** | **0.038 *** |
| Fth1 | **0.795027307** | **0.885491946** | **1.237648751** | **-0.34126045** | **[-0.45, -0.23]** | **-6.66941984** | **-2.240126687** | **<0.001 ***** |
| Gpx1 | 0.943156154 | 4.231170533 | 4.333988904 | -0.094148522 | [-0.19, 0.0054] | -1.96698414 | -0.765679412 | 0.063 |
| Gpx3 | 1.042421308 | 6.319821809 | 6.302708767 | 0.051174178 | [-0.066, 0.17] | 0.90892046 | 0.080019892 | 0.374 |
| Gpx4 | **0.927511168** | **4.280015062** | **4.39421687** | **-0.114201809** | **[-0.22, -0.0088]** | **-2.25287873** | **-0.817357508** | **0.035 *** |
| Gpx7 | **0.859932249** | **7.080293789** | **7.319214963** | **-0.220812757** | **[-0.3, -0.14]** | **-5.69848621** | **-1.254742874** | **<0.001 ***** |
| Gpx8 | 0.945226228 | 7.161568203 | 7.297933339 | -0.098038695 | [-0.21, 0.018] | -1.75933826 | -0.678314187 | 0.093 |
| Gsr | 0.928008246 | 4.902904262 | 5.029061933 | -0.118051506 | [-0.27, 0.033] | -1.62939086 | -0.618977237 | 0.118 |
| Gss | 1.054673927 | 7.831836071 | 7.784705113 | 0.053467933 | [-0.089, 0.2] | 0.78171228 | 0.199406357 | 0.443 |
| Gstk1 | **0.947222059** | **7.970946382** | **8.084491299** | **-0.113544922** | **[-0.21, -0.017]** | **-2.4442897** | **-0.936663645** | **0.023 *** |
| Gstm1 | **1.239027344** | **4.837696463** | **4.517984723** | **0.319711741** | **[0.21, 0.43]** | **6.2323724** | **2.359844659** | **<0.001 ***** |
| Hadh | 1.051150377 | 6.105567557 | 6.105938862 | 0.062270747 | [-0.023, 0.15] | 1.51921105 | -0.002144386 | 0.144 |
| Hif1α | **1.07954192** | **4.788986212** | **4.740251492** | **0.105097599** | **[0.037, 0.17]** | **3.2188656** | **0.264951672** | **0.004 **** |
| Hmox1 | **1.449373017** | **7.866616573** | **7.338318394** | **0.528298179** | **[0.098, 0.96]** | **2.55150135** | **0.892309076** | **0.019 *** |
| Idh1 | **1.076292279** | **3.495301062** | **3.366418408** | **0.110536107** | **[0.044, 0.18]** | **3.48087495** | **1.107746846** | **0.002 **** |
| Ndufv2 | **0.916589123** | **1.717166271** | **1.892093609** | **-0.149004267** | **[-0.26, -0.04]** | **-2.84444403** | **-1.282430717** | **0.010 **** |
| Nos1 | **0.855651816** | **6.621599967** | **6.809994464** | **-0.205929901** | **[-0.37, -0.038]** | **-2.54461902** | **-0.809964216** | **0.019 *** |
| Nqo1 | 0.9992977 | 9.003167095 | 8.994080685 | 0.00908641 | [-0.15, 0.17] | 0.11965384 | 0.047193945 | 0.906 |
| Parp1 | **0.856514073** | **5.799365749** | **6.081332921** | **-0.23291455** | **[-0.33, -0.13]** | **-4.86369494** | **-1.631458778** | **<0.001 ***** |
| Por | 1.013099808 | 5.075462475 | 5.065259503 | 0.016939935 | [-0.11, 0.14] | 0.28135383 | 0.044664301 | 0.781 |
| Prdx1 | **0.880579973** | **1.937549343** | **2.147491406** | **-0.189605261** | **[-0.27, -0.11]** | **-4.96895532** | **-1.384117525** | **<0.001 ***** |
| Prdx2 | 0.970208002 | 2.842361711 | 2.918415021 | -0.056043339 | [-0.13, 0.017] | -1.60484975 | -0.800311068 | 0.123 |
| Prdx3 | 0.965858205 | 5.155521526 | 5.270882557 | -0.060140847 | [-0.14, 0.024] | -1.4897351 | -0.777692151 | 0.151 |
| Prdx4 | 1.068857575 | 5.56208306 | 5.467628621 | 0.093253199 | [-0.012, 0.2] | 1.83476927 | 0.514319493 | 0.081 |
| Prdx5 | 0.927347222 | 3.195616283 | 3.351269291 | -0.139197767 | [-0.29, 0.0095] | -1.94721249 | -0.803815341 | 0.065 |
| Prdx6 | 1.005072792 | 4.149100501 | 4.186116932 | -0.007108367 | [-0.12, 0.11] | -0.12634461 | -0.189600756 | 0.901 |
| Sod1 | **0.901691884** | **1.902865607** | **1.824410199** | **-0.149531093** | **[-0.24, -0.059]** | **-3.4413604** | **0.061911477** | **0.002 **** |
| Sod2 | **1.207819919** | **3.61796634** | **3.289964055** | **0.255499925** | **[0.034, 0.48]** | **2.3950507** | **0.33073948** | **0.026 *** |
| Sod3 | **1.889785622** | **5.323452757** | **3.869291377** | **0.770766111** | **[0.24, 1.3]** | **3.04419683** | **0.347067498** | **0.006 **** |
| Srxn1 | **0.819579439** | **5.171401602** | **5.373212002** | **-0.288052655** | **[-0.38, -0.19]** | **-6.19069043** | **-0.214769087** | **<0.001 ***** |
| Txnip | 0.978655031 | 4.864162706 | 4.711002682 | -0.075026972 | [-0.41, 0.26] | -0.47189974 | 0.149759844 | 0.642 |
| Txnrd1 | 1.055447021 | 4.887084967 | 4.611986302 | 0.076317094 | [-0.0097, 0.16] | 1.84546982 | 0.251331266 | 0.079 |
| Txnrd3 | **1.904100738** | **7.398331771** | **6.359954213** | **0.83982495** | **[0.49, 1.2]** | **5.04212011** | **0.756234142** | **<0.001 ***** |

***Table S1. Full statistical summary of RT-qPCR data.***

Alphabetical list of all the genes tested. All tests were performed on DCq values of which the means of normoxic (N) and hypoxic (IH) and differences between both are indicated here. Test statistics (t), confidence intervals (CI 95), effect size (Hedge g) and p values were computed and are summarized in the table. Fold change is the result of the $2^{-\Delta\Delta Cq}$calculation. Bold text represents statistically significant results, which are discussed in the text.
