# Supplementary figures and images for: Intermittent hypoxia in a mouse model of apnea of prematurity leads to a retardation of cerebellar development and long-term functional deficits
Source: Cell Biosci. 2022 Sep 6;12:148. doi: 10.1186/s13578-022-00869-5 (PMC9450451; doi:10.1186/s13578-022-00869-5)

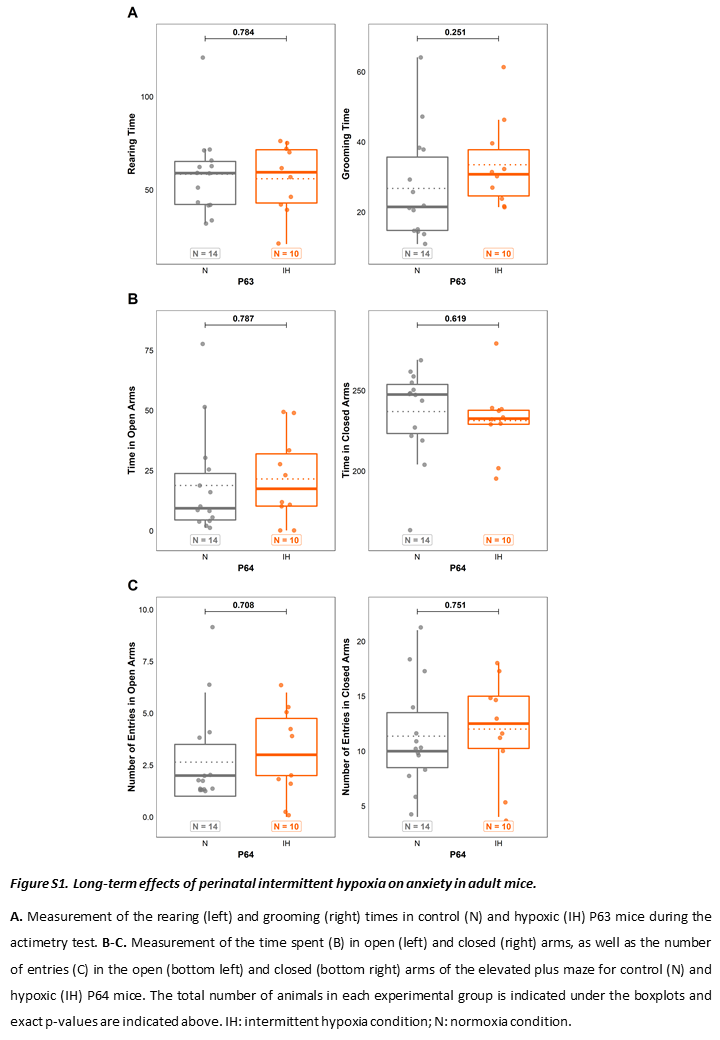

Supplement: Supplementary file 2 — Additional file 2: Figure S1. Long-term effects of potential intermittent hypoxia on anxiety in adult mice. [file 13578_2022_869_MOESM2_ESM.png]

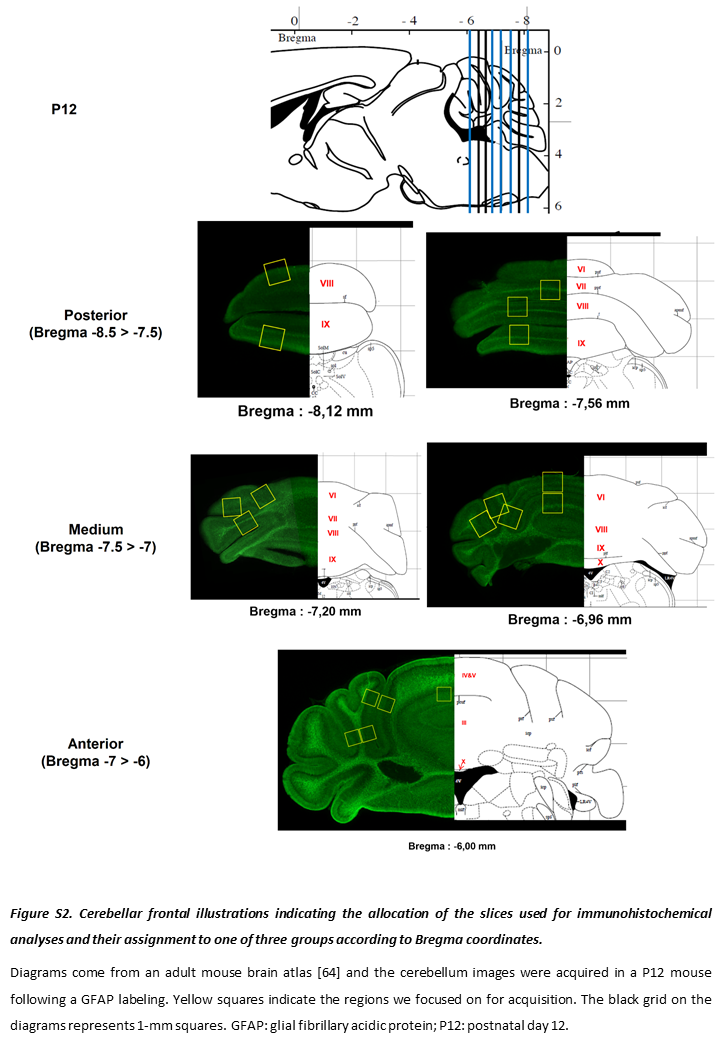

Supplement: Supplementary file 4 — Additional file 4: Figure S2. Cerebellar frontal illustrations indicating the allocation of the slices used for immunuhistochemical analyses and their assignment to one of three groups according to Bregma coordinates. [file 13578_2022_869_MOESM4_ESM.png]
