# Supplementary material for: Intermittent hypoxia in a mouse model of apnea of prematurity leads to a retardation of cerebellar development and long-term functional deficits
Source: Cell Biosci. 2022 Sep 6;12:148. doi: 10.1186/s13578-022-00869-5 (PMC9450451; doi:10.1186/s13578-022-00869-5)
Supplement: Supplementary file 3 — Additional file 3: Table S2. List of primers used for RT-qPCR experiments and their corresponding sequences. [file 13578_2022_869_MOESM3_ESM.docx]

| **Panel** | **Gene** | **Gene name** | **NCBI reference** | **Forward primer** | **Reverse primer** |
| --- | --- | --- | --- | --- | --- |
| ***Apoptosis*** | Bax | BCL2-associated X protein | NM_007527.3 | GTGAGCGGCTGCTTGTCT | GGTCCCGAAGTAGGAGAGGA |
| ***Apoptosis*** | Bcl2 | B-cell CLL/lymphoma 2 | NM_009741.4 | GTACCTGAACCGGCATCTG | GGGGCCATATAGTTCCACAA |
| ***Apoptosis*** | Casp3 | caspase 3, apoptosis-related cysteine peptidase | NM_001284409.1 | GAGGCTGACTTCCTGTATGCTT | AACCACGACCCGTCCTTT |
| ***Apoptosis*** | Casp9 | caspase 9, apoptosis-related cysteine peptidase | NM_001277932.1 | TGCAGTCCCTCCTTCTCAG | GCTTTTTCCGGAGGAAGTTAAA |
| ***Apoptosis*** | Hif1α | hypoxia inducible factor 1, alpha subunit | NM_010431.2 | GTGCACCCTAACAAGCCGGGG | CCGTGCAGTGAAGCACCTTCCA |
| ***Apoptosis*** | Parp1 | poly (ADP-ribose) polymerase 1 | NM_007415.2 | CTATAGTCTTCTCAGGGGTG | TCTCTGTCAACCACCTTAAT |
| ***ROS Production*** | Cox4i1 | cytochrome c oxidase subunit IV isoform 1 | NM_009941.2 | TGTGCCTTCGAGCACATGGGAG | GGCAAGGGGTAGTCACGCCG |
| ***ROS Production*** | Fth1 | ferritin, heavy polypeptide 1 | NM_010239.2 | ACCGTGTCCCAGGGTGTGCTT | ACCGTGTCCCAGGGTGTGCTT |
| ***ROS Production*** | Hadh | hydroxyacyl-CoA dehydrogenase | NM_008212.4 | AGGCTACACGAGCGAGGCGA | ACGGACCCATGGGATACCCAGC |
| ***ROS Production*** | Hmox1 | heme oxygenase (decycling) 1 | NM_010442.2 | TCGAGCATAGCCCGGAGCCT | AATCCTGGGGCATGCTGTCGG |
| ***ROS Production*** | Idh1 | isocitrate dehydrogenase 1 (NADP+), soluble | NM_001111320.1 | CATTCTGGGTGGCACTGTCTT | TATGCATGTCGGCCAATGA |
| ***ROS Production*** | Ndufv2 | NADH dehydrogenase (ubiquinone) flavoprotein 2 | NM_001278415.1 | GCTATGAACAAGGTGGCTGAA | TCCCAACTGGCTTTCGATTA |
| ***ROS Production*** | Nos1 | nitric oxide synthase 1 | NM_008712.2 | CCTGGAAGGATGGAAGAAACG | CAGGCTGCTTGGAGCAAAA |
| ***ROS Production*** | Nqo1 | NAD(P)H dehydrogenase, quinone 1 | NM_008706.5 | TCCCAGGTTGCCCACATTCCCA | TCCAGGGCAAGCGACTCATGGTC |
| ***ROS Production*** | Por | P450 (cytochrome) oxidoreductase | NM_008898.1 | GGCCCACAAGGTCTATGTTC | TCTTTGGCCATATTTCGAGC |
| ***Antioxidant Response*** | Cat | catalase | NM_009804.2 | GGAGGCGGGAACCCAATA | CAAAGTGTGCCATCTCGTCAGT |
| ***Antioxidant Response*** | Gpx1 | glutathione peroxidase 1 | NM_008160.6 | ACACCAGGAGAATGGCAAGAA | AACAATGTAAAATTGGGCTCGAA |
| ***Antioxidant Response*** | Gpx3 | glutathione peroxidase 3 | NM_008161.3 | TCAAAGAACTGAATGCACTACAAGAA | TTCTCGCCTGGCTCCTGTT |
| ***Antioxidant Response*** | Gpx4 | glutathione peroxidase 4 | NM_001037741.3 | GGGCCGTCTGAGCCGCTTAC | TCGCGGGATGCACACATGGT |
| ***Antioxidant Response*** | Gpx7 | glutathione peroxidase 7 | NM_024198.3 | GCCTTCAAGTACCTAACCCAGAC | TGCTCTGTAATACGGGGCTT |
| ***Antioxidant Response*** | Gpx8 | glutathione peroxidase 8 | NM_027127.2 | AGATATTTGCAGTTTTGCTCTCTATGG | GGTTCTCGGCTTCAGGAATTT |
| ***Antioxidant Response*** | Gsr | glutathione reductase | NM_010344.4 | GCCTTTACCCCGATGTATCA | AATGCCAACCACCTTTTCCT |
| ***Antioxidant Response*** | Gss | glutathione synthetase | NM_001291111.1 | CAGCTGTGCACCGACACGTTCT | GGCCAGTCCCTTGCTGGGGT |
| ***Antioxidant Response*** | Gstk1 | glutathione S-transferase kappa 1 | NM_029555.2 | AAGACAGCGGAAACCAACCA | AAGAACTGCTTCAGGAGAGGAATC |
| ***Antioxidant Response*** | Gstm1 | glutathione S-transferase mu 1 | NM_010358.5 | GGTCAGTCCTGCTGAAGCCAGTTT | GGATCGGGTGTGTCAGTCCGC |
| ***Antioxidant Response*** | Prdx1 | peroxiredoxin 1 | NM_011034.4 | GTGAGACCTGTGGCTCGAC | TGTCCATCTGGCATAACAGC |
| ***Antioxidant Response*** | Prdx2 | peroxiredoxin 2 | NM_011563.5 | GGCTCTTGCTCACGCAGT | GAAGGCACCATCCACCAC |
| ***Antioxidant Response*** | Prdx3 | peroxiredoxin 3 | NM_007452.2 | CTTTAGCACCAGTTCCTCTTTCCA | GACTCAGCTCTTTGAACTCTCCATT |
| ***Antioxidant Response*** | Prdx4 | peroxiredoxin 4 | NM_016764.4 | GACGAGACACTGCGTTTGG | GCAGACTTCTCCATGCTTGTC |
| ***Antioxidant Response*** | Prdx5 | peroxiredoxin 5 | NM_012021.2 | CGAGTCCTGGGCTGCAAA | CACACTCCCAACCTGCTTCTTT |
| ***Antioxidant Response*** | Prdx6 | peroxiredoxin 6 | NM_007453.3 | CCACCACGGGCAGGAA | GGGAACTACCATCACGCTCTCT |
| ***Antioxidant Response*** | Sod1 | superoxide dismutase 1 | NM_011434.1 | AAGCGGTGAACCAGTTGTGTT | CTGCACTGGTACAGCCTTGTGTA |
| ***Antioxidant Response*** | Sod2 | superoxide dismutase 2 | NM_013671.3 | GCGCTGGAGCCACACATTA | GGTGGCGTTGAGATTGTTCA |
| ***Antioxidant Response*** | Sod3 | superoxide dismutase 3 | NM_011435.3 | GGGGAGGCAACTCAGAGG | TGGCTGAGGTTCTCTGCAC |
| ***Antioxidant Response*** | Srxn1 | sulfiredoxin 1 | NM_029688.5 | AGGGGCTTCTGCAAACCTA | TGGCATAGCTACCTCACTGCT |
| ***Antioxidant Response*** | Txnip | thioredoxin interacting protein | NM_001009935.2 | GTGGCCGGACGGGTAATAGT | CCTTGCATCCACAGGACCTT |
| ***Antioxidant Response*** | Txnrd1 | thioredoxin reductase 1 | NM_001042513.1 | ACCGTGGGCGTGAAGATAAA | GATGTCACCGATGGCGTAGAT |
| ***Antioxidant Response*** | Txnrd3 | thioredoxin reductase 3 | NM_001178058.1 | GTGAACGTAGGCTGTATTCCAAAGA | TTGTGCTTCACCTGCTGGTTATA |
| ***Housekeeping Genes*** | Hsp90ab1 | heat shock protein 90 alpha, class B member 1 | NM_008302.3 | CAGAAATTGCCCAGCTCATGT | CCGTCAGGCTCTCATATCGAA |
| ***Housekeeping Genes*** | Ppia | peptidylprolyl isomerase A | NM_008907.1 | CCACTGTCGCTTTTCGCCGC | TGCAAACAGCTCGAAGGAGACGC |
| ***Housekeeping Genes*** | Ywhaz | Tyrosine 3-Monooxygenase/Tryptophan 5-Monooxygenase Activation Protein Zeta | NM_011740.3 | AGGACCTAAAAGGGTCGGTCA | CGGGGTTTCCTCCAATCACT |

***Table S2. List of primers used for RT-qPCR experiments and their corresponding sequences.*** Table providing the list of genes tested, its full name and National Center for Biotechnology Information reference number. Genes are divided into three panels, namely "Apoptosis", "ROS production" and "Antioxidant response". Primer sequences indicated were designed on the Primer Express software and ordered from Integrated DNA Technologies.
